# Tetrabromobisphenol A: MAK Value Documentation – Translation of the German version from 2023

**DOI:** 10.34865/mb7994e10_4or

**Published:** 2025-12-22

**Authors:** Andrea Hartwig, MAK Commission

**Affiliations:** 1 Institute of Applied Biosciences. Department of Food Chemistry and Toxicology. Karlsruhe Institute of Technology (KIT) Adenauerring 20a, Building 50.41 76131 Karlsruhe Germany; 2 Permanent Senate Commission for the Investigation of Health Hazards of Chemical Compounds in the Work Area. Deutsche Forschungsgemeinschaft, Kennedyallee 40, 53175 Bonn, Germany. Further information: Permanent Senate Commission for the Investigation of Health Hazards of Chemical Compounds in the Work Area | DFG

**Keywords:** tetrabromobisphenol A, carcinogenicity, uterine adenocarcinoma, hepatoblastoma, hemangiosarcoma, endocrine effects, thyroid hormones, air

## Abstract

The German Senate Commission for the Investigation of Health Hazards of Chemical Compounds in the Work Area (MAK Commission) has evaluated the data for tetrabromobisphenol A [79-94-7] considering all toxicological end points. Relevant studies were identified from a literature search, epidemiological studies of tetrabromobisphenol A are not available. After repeated administration by gavage, the target organs of toxicity were the endocrine system in male and female rats with decreased total T4 concentrations and the kidneys in male mice with cytoplasmic changes in the tubules. Tetrabromobisphenol A induces uterine tumours in female Wistar-Han rats and hepatoblastomas and haemangiosarcomas in male B6C3F1/N mice. Due to its non-genotoxic mechanism of action, it is assumed that a NAEL (no adverse effect level) can be determined for carcinogenic effects. Therefore, tetrabromobisphenol A could be classified in Carcinogen Category 4. However, there are no data that can be used to establish a NOAEL for atypical hyperplasia (preneoplasia) in the endometrium, to clarify whether the observed uterine tumours are specific to the strain, and to further characterize the mechanism of tumourigenesis. Therefore, a maximum concentration at the workplace (MAK value) cannot be established and tetrabromobisphenol A has been classified in Carcinogen Category 2 and given the footnote “Prerequisite for Category 4 in principle fulfilled, but insufficient data available for the establishment of a MAK or BAT value”. Tetrabromobisphenol A is not mutagenic in bacteria or clastogenic in mammalian cells. In vivo data do not provide evidence of genotoxic effects in soma cells, even at concentrations that cause systemic toxicity. In vivo studies suggest that transdermal uptake may be relevant. As tetrabromobisphenol A has been classified as a Category 2 carcinogen and no threshold for the carcinogenic effects can be established at present, the substance has provisionally been designated with “H”. Data for humans, animals and from in vitro studies show no sensitizing potential.

**Table:** 

**MAK value**	**–**
**Peak limitation**	**–**
	
**Absorption through the skin (2022)**	**H**
**Sensitization**	**–**
**Carcinogenicity (2022)**	**Category 2** ^a)^
**Prenatal toxicity**	**–**
**Germ cell mutagenicity**	**–**
	
**BAT value**	**–**
	
Synonyms	2,2-bis(3,5-dibromo-4-hydroxyphenyl)propane 4,4′-isopropylidenebis(2,6-dibromophenol) 2,2′,6,6′-tetrabromobisphenol A 2,2′,6,6′-tetrabromo-4,4′-isopropylidenebisphenol
Chemical name	2,6-dibromo-4-[2-(3,5-dibromo-4-hydroxyphenyl)propan-2-yl]phenol
CAS number	79-94-7
Structural formula	Structural formula of tetrabromobisphenol A. 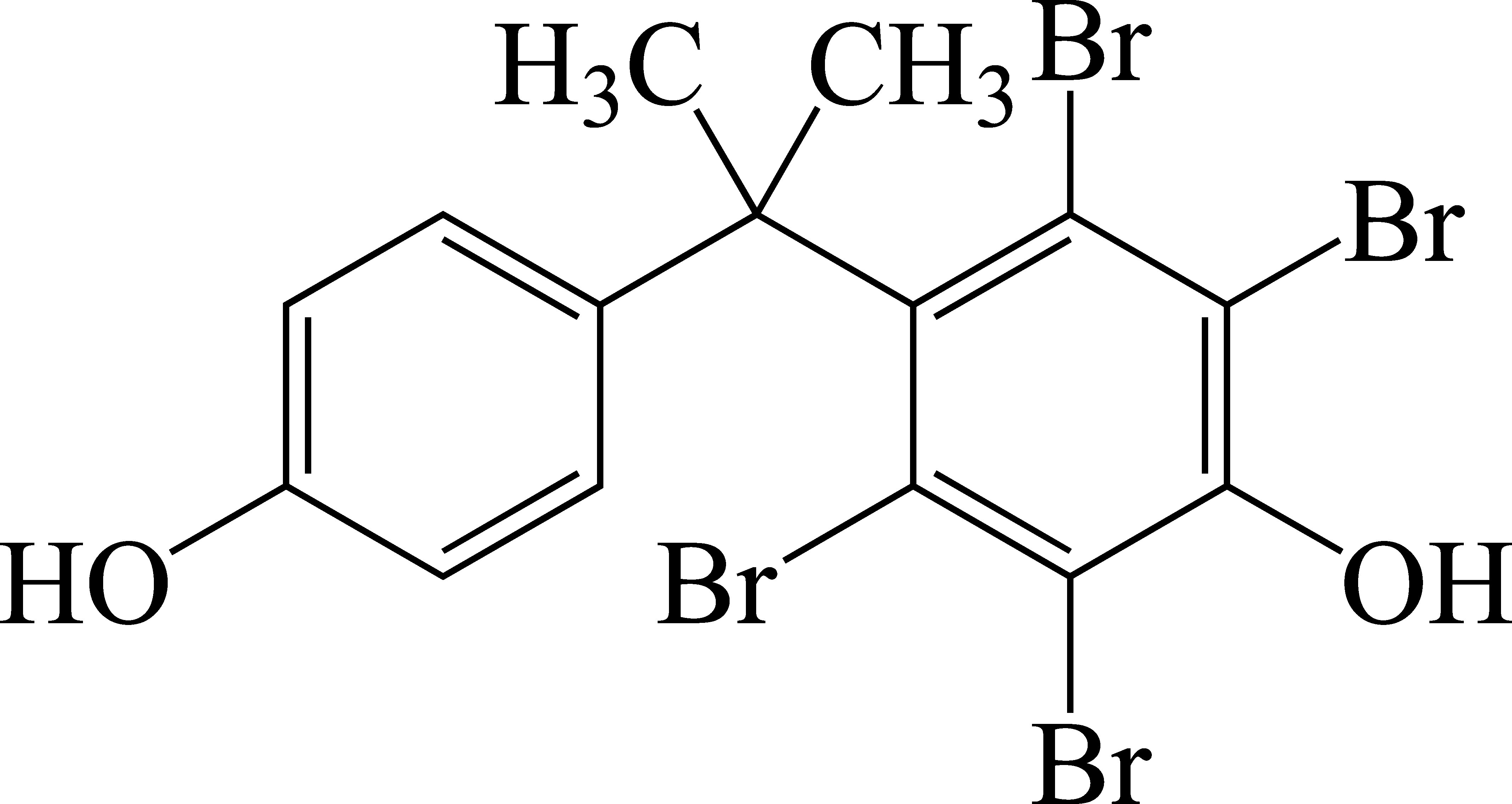
Molecular formula	C_15_H_12_Br_4_O_2_
Molar mass	543.88 g/mol
Melting point	179–181 °C (IFA 2019)
Boiling point	decomposes at 316 °C (ECHA 2019)
Vapour pressure at 20 °C	< 1.19 × 10^–7^ hPa (experimental; ECHA 2019)
log K_OW_ at 25 °C	5.9 (no data for pH; ECHA 2019)
Solubility at 25 °C	0.24 mg/l water (IFA 2019)
pKa value at 20 °C	9.4 (ECHA 2019)
**1 ml/m^3^ (ppm) ≙ 22.57 mg/m^3^**	**1 mg/m^3^ ≙ 0.0443 ml/m^3^ (ppm)**

^a)^ Prerequisite for Category 4 in principle fulfilled, but insufficient data available for the establishment of a MAK or BAT value

Tetrabromobisphenol A is used as a flame retardant in circuit boards, in housings for electrical and electronic equipment and in plastics (EFSA 2011; EU 2006; NTP 2014).

Inhalation exposure to tetrabromobisphenol A varies by several orders of magnitude across the industry sectors. The highest concentrations in air were found in plastics production with 8-hour time-weighted averages of up to 12.2 mg/m^3^ during loading and mixing processes (EU 2006).

## 1 Toxic Effects and Mode of Action

Tetrabromobisphenol A was carcinogenic in animal studies. Female Wistar Han rats given gavage doses of tetrabromobisphenol A for 2 years were found to have an increased incidence of uterine adenocarcinomas at doses of 500 mg/kg body weight and day and above. Malignant mixed Müllerian tumours, otherwise a very rare form of tumour, were induced in a few isolated cases. The tumour incidence in male rats was not increased with statistical significance after exposure to tetrabromobisphenol A. After male B6C3F1/N mice were exposed to the substance for 2 years, an increase in hepatoblastomas was found at doses of 250 mg/kg body weight and day and above and haemangiosarcomas (all organs) were observed at 500 mg/kg body weight and day. A substance-related increase in tumour incidences was not determined in female mice.

In rats, oral administration for 28 days with the feed and for 13 weeks by gavage led to a decrease in the total thyroxine (T4) concentrations at 100 mg/kg body weight and day and above, disrupting the homeostasis of thyroid hormone levels. Male animals are more susceptible to this effect than female animals. After tetrabromobisphenol A was given to male mice by gavage for 14 weeks, liver weights were increased and histopathological changes in the renal tubules were observed at doses of 500 mg/kg body weight and day and above.

In prenatal developmental toxicity studies with gavage administration in rats from gestation days 0 to 19, neither maternal toxicity, developmental toxicity nor teratogenicity was observed up to the highest dose tested of 2500 mg/kg body weight and day.

Tetrabromobisphenol A did not cause irritation of the skin in rabbits and rats or irritation of the eyes in rabbits.

There was no evidence that would suggest that tetrabromobisphenol A causes sensitizing effects.

On the basis of the data available, the substance is not regarded as genotoxic.

## 2 Mechanism of Action

### 2.1 Receptor-mediated effects

#### 2.1.1 Endocrine effects

Numerous studies, particularly those that included reporter gene assays, found no evidence that tetrabromobisphenol A activates the oestrogen receptor (ER) (Hamers et al. 2006; Judson et al. 2015; Molina-Molina et al. 2013; Riu et al. 2011 a, b; Wikoff et al. 2016). Other studies, however, did detect slight interaction of the substance with the ER that was described as minimal oestrogenic (Bermudez et al. 2010; Kitamura et al. 2002; Li et al. 2010; Olsen et al. 2003; Samuelsen et al. 2001) or antagonistic (Kitamura et al. 2005 b). According to a complex model using ToxCast data from 18 in vitro tests, tetrabromobisphenol A was inactive as an oestrogen receptor agonist or antagonist (IARC 2018; Judson et al. 2015). To evaluate the agonistic and antagonistic effects on the ER, the model integrated the end points receptor binding, receptor dimerization, chromatin binding of the mature transcription factor, transcriptional activation and ER-dependent cell proliferation (Judson et al. 2015).

In the Alamar Blue assay, tetrabromobisphenol A increased cell proliferation in the human ovarian carcinoma cell line OVCAR-3 and the granulosa cell line KGN at concentrations of 10 nM and above. The proliferation levels were 1.2 to 1.3 times the control levels. Cell proliferation was prevented by an inhibitor of the selective G protein-coupled oestrogen receptor (GPER, GPR30, gene: GPER1; Barton et al. 2018) (Hoffmann et al. 2017 b). The GPER antagonist G15 was added in a concentration of 1 μM; at this concentration, the antagonist weakly activates ERα (Dennis et al. 2011). Therefore, the antagonist used was not specific for GPER. However, clear evidence of possible effects induced by tetrabromobisphenol A at this receptor, such as findings from gene knockdown/knockout studies, is not available.

Tetrabromobisphenol A promoted the expression of GPER in 3T3-L1 preadipocytes of mice (Chappell et al. 2018). In a competitive binding assay in SKBR3 breast cancer cells, low affinity to GPER was determined at concentrations of 100 µM and above (Cao et al. 2017). There are no studies available that investigated the effects of tetrabromobisphenol A on GPER in endometrial cells. Oestrogen-like effects are mediated also via non-genomic signalling pathways, namely membrane-coupled GPER (Watson et al. 2010).

GPER is a transmembrane receptor with 7 domains (Barton et al. 2018; Filardo and Thomas 2012). The receptor is primarily located intracellularly on the endoplasmic reticulum, but also on the Golgi apparatus and on the cell nucleus (Gaudet et al. 2015; Prossnitz et al. 2008; Revankar et al. 2005). The receptor is expressed also on the cell surface of certain tissues such as oocytes, the uterine epithelium and hippocampal neurons (Barton et al. 2018; Gaudet et al. 2015). GPER is expressed in many different human tissues such as the mammary glands, placenta, bone marrow, thymus, skeletal muscles, brain, blood vessels, lungs, liver, intestines and reproductive organs (Jacenik et al. 2016), but particularly in the endometrial cells of women (EMBL-EBI 2020 a; Plante et al. 2012) and in the uterus of women and female rats (EMBL-EBI 2020 b). Increased expression was found also in the human endometrial cancer cell lines HEC1A and Ishikawa (Plante et al. 2012; Vivacqua et al. 2006) as well as in tissue samples of atypical endometrial hyperplasia and type I endometrial carcinomas (Plante et al. 2012; Xie et al. 2017). The signal chains triggered by the receptor are described as “pregenomic” events (second messenger and protein/lipid kinase activation) that occur within minutes and lead to gene transactivation. ER and GPER vary in their binding affinity for different oestrogens. Oestrogen antagonists may act also as GPER agonists (Filardo and Thomas 2012).

Fertility and sexual development were not impaired in GPER1 knockout mice (Otto et al. 2009; Zimmerman et al. 2016). However, the receptor regulates physiological processes in the intestinal tract, nervous and immune systems as well as in the kidneys and circulatory system (Barton et al. 2018; Filardo and Thomas 2012; Jacenik et al. 2019; Prossnitz and Barton 2009). GPER seems to play an additional, modulating role in cell proliferation and cell migration in the endometrium, while ERα is imperative for these functions (Dennis et al. 2009; Gibson et al. 2020; Prossnitz and Barton 2009).

Extensive research has shown the effects of GPER on cell proliferation and tumour development (Barton et al. 2018; Jacenik et al. 2016, 2019; Zimmerman et al. 2016). For example, the receptor mediated the proliferative effects induced by 17β-oestradiol and hydroxytamoxifen in the endometrium in the human endometrial cancer cell lines HEC1A and Ishikawa (Vivacqua et al. 2006). Evidence was found of the transactivation of growth factor receptors such as EGFR (epidermal growth factor receptor) (Barton et al. 2018; Jacenik et al. 2016). The findings reveal also complex interactions between GPER and HER2 (human epidermal growth factor receptor 2) as well as IGF-IR (insulin-like growth factor-I receptor) and how these may contribute to the progression of various tumour types (Lappano et al. 2013). Other research has shown that tumour-suppressing effects in ovarian and mammary gland tumours are mediated via GPER (Hernández-Silva et al. 2020; Ignatov et al. 2013).

Compared with bisphenol A, tetrabromobisphenol A stimulated OVCAR-3 and KGN cell proliferation mediated by GPER with lower potency (140% and 120%, respectively, of the controls after 48 hours at 50 nM) (Hoffmann et al. 2017 b). Whether bisphenol A also causes uterine tumours in Wistar Han rats (see also Section 2.8) has not been determined. Bisphenol A did not induce uterine tumours in F344 rats in a carcinogenicity study (NTP 1982).

As tetrabromobisphenol A stimulates proliferation in ovarian cells (Hoffmann et al. 2017 b), it may have this effect also in endometrial cells because these express GPER. It is thus plausible that the substance may induce promoting effects in the endometrium via GPER. However, as described above, a gene knockdown/knockout assay has not been performed and there is no evidence that tetrabromobisphenol A activates the GPER.

Although 2 studies found that tetrabromobisphenol A causes weak to moderate antagonistic effects on the androgen receptor (Beck et al. 2016; Christen et al. 2010), no other study found signs of interaction with the androgen receptor (Hamers et al. 2006; Kitamura et al. 2005 b; Li et al. 2010; Molina-Molina et al. 2013). According to data from the ToxCast database, the substance has an antagonistic effect on the androgen receptor (US EPA 2021 b). One study found that tetrabromobisphenol A has strong antagonistic effects on the progesterone receptor (PR) (Li et al. 2010), while another did not find any signs of interaction with the PR (Hamers et al. 2006). Tetrabromobisphenol A was a potent agonist of the human peroxisome proliferator-activated receptor γ (PPARγ) (Honkisz and Wójtowicz 2015 b; Riu et al. 2011 a, b) and a partial agonist of murine PPARγ (Chappell et al. 2018; Watt and Schlezinger 2015) .

Numerous studies described direct and indirect changes in the activation of the thyroid hormone receptor that are caused by tetrabromobisphenol A (Butt et al. 2011; Freitas et al. 2011; Guyot et al. 2014; Hamers et al. 2006; Hofmann et al. 2009; Jugan et al. 2007; Kitamura et al. 2002, 2005 a, b; Lévy-Bimbot et al. 2012; Meerts et al. 2000; Oka et al. 2013; Sun et al. 2009; Terasaki et al. 2011; Yamada-Okabe et al. 2005); however, the effects of this interaction depend on various variables (IARC 2018). Several in vitro studies reported that the binding affinity of tetrabromobisphenol A to human transthyretin (TTR), a T4 transport protein in the plasma, is stronger than that of the natural ligand T4 (Chi et al. 2020; Hamers et al. 2006; Iakovleva et al. 2016; Meerts et al. 2000). As a result of this stronger affinity, tetrabromobisphenol A may replace T4 on TTR, which would result in an increased depletion of free T4 and decreased total T4 concentrations (Hamers et al. 2006; Meerts et al. 2000). The 50% inhibitory concentration (IC_50_) in the TTR binding assay was 0.031 µM. The binding affinity of tetrabromobisphenol A to TTR was 1.6 times that of T4 (Hamers et al. 2006). In another assay with human TTR, an IC_50_ of 0.0077 µM was determined and the binding affinity was 10.6 times that of T4 (Meerts et al. 2000). In pregnant Wistar rats given ^14^C-tetrabromobisphenol A in a gavage dose of 5 mg/kg body weight and day from gestation days 10 to 16, no effects on the total or free concentrations of triiodothyronine (T3) or T4 were observed in the dams and foetuses. Binding of tetrabromobisphenol A to TTR was not observed. Only 0.83% of the radioactivity was recovered in the maternal tissue (Meerts et al. 1999). However, because of the short exposure period and low dose, it is unlikely that the substance would bind to TTR. Therefore, the findings of the study cannot be used as evidence that tetrabromobisphenol A does not bind to TTR in vivo.

The substance led to a decrease in the total T4 concentration in serum in several studies with rats (Choi et al. 2011; Cope et al. 2015; NTP 2014; Osimitz et al. 2016; van der Ven et al. 2008). The lowest effective dose was 100 mg/kg body weight and day in studies with administration of the substance for 28 days with the feed (van der Ven et al. 2008) or by gavage for 13 weeks (Osimitz et al. 2016), about 18 weeks (Cope et al. 2015) and 2 years (NTP 2014).

Tetrabromobisphenol A concentrations of 1 µM and above stimulated cell growth and the release of growth hormones in a rat pituitary GH3 cell line responsive to thyroid hormones. This suggests that the substance is a thyroid hormone agonist (Ghisari and Bonefeld-Jorgensen 2005; Kitamura et al. 2002, 2005 b; Strauss and van Ravenzwaay 2017).

#### 2.1.2 Other receptor-mediated effects

In many cases, tetrabromobisphenol A increased the expression and secretion of apelin; this was described as a PPARγ-dependent effect. After exposure to tetrabromobisphenol A in the concentration range between 1 and 100 nM, the increase in apelin expression was statistically significant, but not dependent on the concentration. The results of PPARγ gene knockout experiments confirmed that tetrabromobisphenol A induces PPARγ-dependent effects (Hoffmann et al. 2017 a). PPARγ modulates lipid homeostasis (Han et al. 2017; Wang et al. 2020). The PPARγ agonistic effects and the formation of haemangiosarcomas in male B6C3F1/N mice are discussed in Section 2.10.

The substance was a weak to moderate activator of hPXR (human pregnane X receptor) with an EC_50_ of 11.97 µM (Molina-Molina et al. 2013). There was no evidence of direct interaction with the Ah (aryl hydrocarbon) receptor, of the activation of Ah receptor-mediated cytochrome P450 (CYP) 1A1 in vitro or in vivo (Behnisch et al. 2003; Brown et al. 2004; Hamers et al. 2006; NTP 2014) or of direct interaction with the glucocorticoid receptor (Beck et al. 2016).

An evaluation of the ToxCast data (US EPA 2021 a) revealed that tetrabromobisphenol A is a substance with many cellular points of action, as evidenced by the large number of active tests. An evaluation carried out by the IARC found that the substance interacted with various nuclear receptors such as PPARγ and PXR, which supports the investigated mechanisms of action for tetrabromobisphenol A (IARC 2018).

### 2.2 Disturbances in hormone metabolism

Tetrabromobisphenol A inhibited the expression of the human recombinant sulfotransferase isoforms SULT1E1 and SULT1A1 that metabolize oestradiol in Salmonella typhimurium with IC_50_ values of 12 to 33 nM (in comparison: bisphenol A: IC_50_ > 10 000 nM; Kester et al. 2002) and the expression of the isoform SULT1E1 in V79 cells with an IC_50_ value of 16 nM (Hamers et al. 2006). According to an X-ray crystallography study (Gosavi et al. 2013) and a QSAR study (Harju et al. 2007), tetrabromobisphenol A binds to the same binding site on SULT1E1 as oestradiol. The findings of the QSAR study show that the substance meets the requirements for potent inhibition of this enzyme (Wikoff et al. 2016). SULT1E1 is an intracellular enzyme. The IC_50_ determined from in vitro studies cannot be extrapolated to the in vivo situation because the intracellular concentration of tetrabromobisphenol A in vitro is not known.

A study found that tetrabromobisphenol A increased aromatase activity (CYP19) (Honkisz and Wójtowicz 2015 a). However, another study did not find evidence of effects on CYP19 activity or on the expression of the genes CYP11A, CYP11B2, CYP17, CYP21, 3βHSD2 (HSD: heterologous superoxide dismutase), 17βHSD1, 17βHSD4 or StAR (steroidogenic acute regulatory protein) (Song et al. 2008). The ToxCast data, on the other hand, support the inhibition of CYP19 (IARC 2018).

Overall, the modulation of steroid biosynthesis is supported by the ToxCast data (IARC 2018).

In vitro, tetrabromobisphenol A inhibited 3,3′-diiodothyronine sulfotransferase activity in the liver microsomes of male rats (IC_50_: 4.3 µM; reference substance pentachlorophenol: IC_50_: 0.005 µM) (Schuur et al. 1998).

### 2.3 Oxidative stress

Numerous in vitro studies that were carried out with hepatocytes from rats, an osteoblast cell line from mice, human neutrophilic granulocytes, cerebellar granular cells from rats, SH-SY5Y neuroblastoma cells and pancreatic cells from rats found that tetrabromobisphenol A promoted the generation of reactive oxygen species. The effective concentrations were in the µM range (Al-Mousa and Michelangeli 2012; Choi et al. 2017; Nakagawa et al. 2007; Reistad et al. 2005, 2007; Suh et al. 2017; Ziemińska et al. 2012, 2017). Mitochondrial dysfunctions were likewise observed (Choi et al. 2017; Nakagawa et al. 2007; Ogunbayo et al. 2008; Suh et al. 2017; Ziemińska et al. 2012, 2017).

In vivo, tetrabromobisphenol A induced oxidative stress in the testes and kidneys of male Sprague Dawley rats, but not in the liver. The biomarker 8-hydroxy-2′-deoxyguanosine was present in higher concentrations after gavage administration of 500 mg/kg body weight and day for 30 days (Choi et al. 2011). In the kidneys of male Sprague Dawley rats, single doses of 200 mg/kg body weight and above increased oxidative stress (increased superoxide dismutase (SOD) activity; at 1000 mg/kg body weight: increased thiobarbituric acid reactive substances (TBARS)). In the 14-day study, however, these parameters remained unchanged up to a dose of 1000 mg/kg body weight and day (Kang et al. 2009). After receiving gavage doses for 7 days, glutathione (GSH) levels were reduced in the liver of female IMP:WIST rats at 750 mg/kg body weight and day and above and malondialdehyde levels were increased in the liver of male rats at 1125 mg/kg body weight and day (Szymańska et al. 2000). A 2,6-dibromobenzosemiquinone radical was found in the bile of male Sprague Dawley rats; this may form superoxides after reacting with oxygen (Chignell et al. 2008).

A review of the ToxCast data supports the conclusion that tetrabromobisphenol A causes oxidative stress (IARC 2018).

### 2.4 Immunosuppressive and immunological effects

Numerous in vitro studies found that tetrabromobisphenol A in the µM range causes immunosuppressive (Almughamsi and Whalen 2016; Anisuzzaman and Whalen 2016; Choi et al. 2017; Hurd and Whalen 2011; Kibakaya et al. 2009; Pullen et al. 2003; Yasmin and Whalen 2018) and pro-inflammatory effects (Arita et al. 2018; Han et al. 2009; Koike et al. 2013; Park et al. 2014; Suh et al. 2017).

Host immunity against respiratory diseases induced by the respiratory syncytial virus (RSV) was decreased in female BALB/c mice infected with RSV after 4-week administration of 1% tetrabromobisphenol A with the feed (about 1200 mg/kg body weight and day; conversion factor 0.12 (subacute) according to EFSA Scientific Committee (2012)) (Watanabe et al. 2010, 2017). After the offspring of BALB/c mice treated with tetrabromobisphenol A were infected with RSV, the resulting pneumonia was more severe. The authors suggest that IL-24 is the key molecule for the observed effect because a higher expression of interleukin 24 (IL-24) was found in the lung tissue of treated animals (Watanabe et al. 2017).

A microarray analysis found that signal pathways and genes associated with the immune system response were repressed in the uterine tissue of Wistar Han rats given tetrabromobisphenol A in a gavage dose of 250 mg/kg body weight and day for 5 days. The following genes were affected: Ccl6, Clca4, Clec4a3, Clec7a, Ctse, Dmbt1, Fcgr1a, Fcgr3a, RT1-DMb and Tlr4. The changes in Clec7a and Ctse expression affected only the proximal uterus, while those in Dmbt1, RT1-DMb and Tlr4 were observed only in the distal uterus (Hall et al. 2017).

In the offspring of Wistar rats given tetrabromobisphenol A doses of up to 3000 mg/kg body weight and day, no effects on the natural killer cells (NK cells) in the spleen were observed (van der Ven et al. 2008). Neither the peripheral NK cells nor the treated parent animals were examined.

### 2.5 Effects on cell proliferation and cell death

In different in vitro systems, tetrabromobisphenol A did not increase cell proliferation or suppressed apoptosis. However, several studies reported an increase in apoptosis (Choi et al. 2017; Honkisz and Wójtowicz 2015 b; Lenart et al. 2017; Ogunbayo et al. 2008; Reistad et al. 2007; Strack et al. 2007; Suh et al. 2017; Szychowski and Wójtowicz 2016).

In a uterotrophic assay with intraperitoneal injection of the substance in ovariectomized B6C3F1 mice on 3 days, the response was only weak at doses of 20 mg/kg body weight and day and above (5 animals/group, relative uterus weights increased 1.2-fold, 1.6-fold at 300 mg/kg body weight and day, no dose dependency; increase by 17β-oestradiol 5-fold at 50 µg/kg body weight and day; Kitamura et al. 2005 b). OECD Test Guideline 440 recommends treating the animals by gavage or subcutaneous injection and testing groups of at least 6 animals. Negative results were obtained in another uterotrophic assay carried out according to OECD Test Guideline 440 in ovariectomized C57BL/6J mice (6 animals/group) with oral and subcutaneous administration of 1000 mg/kg body weight and day for 7 days. The positive control 17α-ethinyloestradiol, given orally at 6 μg/kg body weight and day or subcutaneously at 0.2 μg/kg body weight and day, increased the uterus weights about 3-fold (Ohta et al. 2012). Therefore, the data suggest that tetrabromobisphenol A has very weak direct oestrogenic effects on proliferation in vivo.

### 2.6 Disturbance of signal cascades and gene regulation systems

Tetrabromobisphenol A was found to impair signal cascades, or complex gene regulation systems, in vitro (Cato et al. 2014; Honkisz and Wójtowicz 2015 b; Lenart et al. 2017; Lévy-Bimbot et al. 2012; Reistad et al. 2005, 2007; Strack et al. 2007). Targets were, among others, MAP (mitogen-activated protein) kinases (Cato et al. 2014; Reistad et al. 2005, 2007; Strack et al. 2007) and caspases (Honkisz and Wójtowicz 2015 b; Lenart et al. 2017).

As described above, a microarray analysis of the uterine tissue of Wistar Han rats detected the reduced expression of genes that are associated with the immune system response (Hall et al. 2017). A study investigated the expression of certain genes in the proximal and distal parts of the uterus and in the liver of female Wistar Han rats that were treated with gavage doses of tetrabromobisphenol A of 250 mg/kg body weight and day for 5 days. The findings revealed an altered expression of genes that code for receptors. Thus, for example, Esr1 (encodes ERα) was activated in the proximal and distal parts of the uterus, while Esr2 (encodes ERβ) was activated in the proximal part of the uterus and silenced in the distal part. Changes were observed also in the genes that are responsible for biosynthesis and for the metabolism of oestrogen in the liver and uterus. For example, Cyp17a1 was activated in the liver and Hsd17b2 (the enzyme responsible for converting oestradiol to its less active form, oestrone) was silenced in the uterus. In addition, exposure altered the expression of genes that are associated with cell division and cell growth (for example, induction of Ccnb1, Ccnb2, Cdk1 in the liver, Igf1 in the liver and uterus). The expression of the genes Nr1i3 (encodes CAR, constitutive androstane receptor) and Nr1i2 (encodes PXR) remained unchanged. After exposure for 5 days, no changes in the oestradiol, total T3 and TSH (thyroid-stimulating hormone) concentrations in serum were observed in comparison with the values obtained in the control group. The total T4 concentration, on the other hand, was reduced with statistical significance. According to the authors, higher concentrations may be found in a healthy or ectopic endometrium if the serum concentrations of oestradiol remain unchanged (Sanders et al. 2016). This finding was obtained by comparing 60 women with endometriosis with 16 healthy women (the proliferation and secretion phases were compared) (Huhtinen et al. 2012). The study findings reported by Sanders et al. (2016) thus support the hypothesis that tetrabromobisphenol A impairs oestrogen homeostasis in female Wistar Han rats.

### 2.7 Neurotoxic effects

In a 2-week gavage study in male C57BL/6 mice, the reduced survival of newly generated cells in the dentate gyrus of the hippocampus at doses of 100 mg/kg body weight and day and above was regarded as an expression of impaired neurogenesis. This effect was dependent on the dose (Kim et al. 2017). However, the mechanism suggested by the authors, the impairment of memory caused by the suppression of the BDNF-CREB signalling pathway (brain-derived neurotrophic factor-cAMP response element-binding protein), cannot be accepted unreservedly because the relevant data were obtained from different animals and only 1 dose group was investigated.

In 10-day-old NMRI mice given a single dose of tetrabromobisphenol A of 11.5 mg/kg body weight, the following protein levels in the brain remained unchanged: CaMKII (calcium/calmodulin-dependent protein kinase II), GAP-43 (growth associated protein 43), synaptophysin and tau (Viberg and Eriksson 2011).

Two modes of action for the induction of developmental neurotoxic effects were identified in vitro in neurospheres in maturing oligodendrocyte progenitor cells. One of the pathways is dependent on thyroid hormone signalling and the other is not. The pathway that is dependent on thyroid hormone (T3) signalling involves the dysregulation of genes that are associated with oligodendrogenesis (for example MBP: myelin basic protein, KLF9: Krueppel-like factor 9 and EGR1: early growth response 1). The other pathway progresses via the disruption of the gene network that regulates cholesterol homeostasis (benchmark concentration with 30% response, BMC_30_ = 1.7 µM). These pathways can be regarded as a novel adverse outcome pathway (AOP) that features hypomyelination as its key event. Human neural progenitor cells were more susceptible to the effects of tetrabromobisphenol A on oligodendrogenesis than the corresponding cells of rats, because tetrabromobisphenol A (up to 1 µM) did not reduce oligodendrocyte differentiation in rats per se and inhibited T3-dependent oligodendrocyte maturation at higher concentrations (BMC_30_ = 0.46 µM) than in human neurospheres (BMC_30_ = 0.06 μM) (Klose et al. 2021).

An effect that may be mediated by thyroid hormone receptors is the modification of the T3-mediated induction of gene expression in the cerebellar cells of mice (Guyot et al. 2014). Several studies that investigated the cerebellar granule cells of rats and human neuroblastoma cells suggest that oxidative stress is involved in this effect (Al-Mousa and Michelangeli 2012; Reistad et al. 2007; Ziemińska et al. 2012, 2017). Additionally, there was evidence of increased apoptosis in the cerebellar granule cells of rats and in primary cultured hippocampal neurons of mouse embryos (Lenart et al. 2017; Reistad et al. 2007; Szychowski and Wójtowicz 2016). In SH-SY5Y human neuroblastoma cells, micromolar amounts of tetrabromobisphenol A increased intracellular calcium concentrations and released β-amyloid peptide (Al-Mousa and Michelangeli 2012). In synaptosomes from rat brains, tetrabromobisphenol A inhibited the uptake of dopamine, glutamate and GABA (γ-amino-*n*-butyric acid) into the cell (Mariussen and Fonnum 2003). Tetrabromobisphenol A altered ABC (ATP-binding cassette) transport at the blood–brain barrier of rat brain capillaries ex vivo (Cannon et al. 2019). Studies of embryonic neural stem cells from rats found that neurodifferentiation was disrupted during the development towards neuronal and glial phenotypes at concentrations of 1 µM and above. However, the generation of neurites and the selection of a neurotransmitter in neuronotypic PC12 cells from rats were not affected up to a concentration of 50 µM (Slotkin et al. 2017).

A broad range of neurotoxic and developmental neurotoxic mechanisms are activated even at low concentrations, affecting various types of cells of the central nervous system as well as many processes, particularly those involved in brain development. Interference with intracellular calcium homeostasis and various neurotransmitters suggest mechanisms that may cause neurotoxic effects in the adult brain. Further studies are necessary for the derivation of a NOAEC (no observed adverse effect concentration).

### 2.8 Development of uterine tumours

In a 2-year carcinogenicity study in Wistar Han rats, gavage doses of tetrabromobisphenol A of 500 mg/kg body weight and day and above increased the incidence of uterine epithelial tumours in the females. The tumours were mainly adenocarcinomas and malignant mixed Müllerian tumours. Atypical hyperplasia of the endometrium occurred as a precursor at doses of 250 mg/kg body weight and day and above (NTP 2014).

Malignant mixed Müllerian tumours are believed to develop from pluripotent cells of the Müllerian duct; both epithelial and stromal cell types can develop simultaneously from these cells. As a result, this form of tumour is characterized by a combination of malignant epithelial and mesenchymal elements (van den Brink-Knol and van Esch 2010). Besides this de novo synthesis, there is sufficient evidence that the sarcomatous component develops from the carcinomatous component via epithelial–mesenchymal transition (conversion theory; Filser 2016). This means that a mixed Müllerian tumour can develop from parts of an adenocarcinoma. This theory is explained also by the authors of the carcinogenicity study published by the NTP (2014). Thus, the epithelial component is considered the predominant element in the histogenesis of malignant mixed Müllerian tumours and the mesenchymal component is the element that is derived from the carcinoma. This hypothesis is supported by the fact that all of the metastases that were detected were carcinomas of epithelial origin (NTP 2014). For this reason, the adenocarcinomas and malignant mixed Müllerian tumours in the uterus of Wistar Han rats are considered together by the Commission in the evaluation of the carcinogenicity study.

Three reviews discussed the development of uterine tumours in Wistar Han rats following exposure to tetrabromobisphenol A (Dunnick et al. 2015; Lai et al. 2015; Wikoff et al. 2016). Important aspects addressed by these reviews are described and discussed below.

There is no evidence that direct oestrogenic effects induced by tetrabromobisphenol A lead to the development of uterine tumours because tetrabromobisphenol A has no or only very slight affinity to the oestrogen receptors ERα and ERβ (Bermudez et al. 2010; Kitamura et al. 2002, 2005 b; Lai et al. 2015; Li et al. 2010; Olsen et al. 2003; Samuelsen et al. 2001; Wikoff et al. 2016) and ER-dependent cell proliferation is induced in vitro only at high concentrations of 10 to 30 µM (Lai et al. 2015; Wikoff et al. 2016).

The ERα receptor was detected by immunohistochemical analysis in the nuclei of uterine tumour cells of rats treated with tetrabromobisphenol A (Harvey et al. 2015). The presence of ERα in the nuclei of uterine tumour cells from rats that were treated with tetrabromobisphenol A proves that the tumour is responsive to oestrogen and suggests that the effects are dependent on oestrogen. This means that tetrabromobisphenol A may indirectly promote the proliferation of epithelial cells in the uterus depending on oestrogen levels.

Tetrabromobisphenol A is a potent inhibitor of SULT1E1, an enzyme that metabolizes oestradiol (Gosavi et al. 2013; Hamers et al. 2006; Harju et al. 2007; Kester et al. 2002; Wikoff et al. 2016). This may lead to increased bioavailability of oestradiol (Borghoff et al. 2016; Wikoff et al. 2016). In Wistar Han rats that were given gavage doses of tetrabromobisphenol A for 28 days, the ratio of tetrabromobisphenol A-sulfate to tetrabromobisphenol A-glucuronide in the plasma, liver and uterus decreased with the dose at 250 mg/kg body weight and day and above. This suggests a limited capacity for sulfation (see Section 3.1.2.2). In this test, tetrabromobisphenol A sulfation was used as a surrogate for oestradiol sulfation because tetrabromobisphenol A and oestradiol bind to the same sites of SULT1E1 (Borghoff et al. 2016). However, the evaluation of the sulfates assessed the total sulfation capacity and not only that catalysed by oestrogen sulfotransferases. The sulfation capacity is maintained also at higher doses, as shown by the increase in sulfoconjugates. However, these findings cannot be used to determine whether the oestrogen-specific SULT1E1 is saturated.

Sult1e1-knockout mice had increased concentrations of oestrogen in the blood (Miller and Flück 2014).

An increased oestradiol–progesterone ratio is known to contribute to the development of uterine tumours in Donryu rats and in women (Wikoff et al. 2016). In women, atypical hyperplasia is usually caused by high oestrogen levels and low progesterone levels (NTP 2014). Female CF-1 mice (in dioestrus) were given a single subcutaneous injection of tetrabromobisphenol A at 1 mg/kg body weight leading to increased excretion of oestradiol with the urine (Pollock et al. 2017). However, only one of the tetrabromobisphenol A studies determined the oestradiol and progesterone concentrations in the blood: After female Wistar Han rats were given gavage doses of 250 mg/kg body weight and day for 5 days, no changes were observed in the oestradiol concentration in serum when compared with the levels found in the controls. According to the authors, higher concentrations in the endometrium are possible if the oestradiol concentrations in the serum remain unchanged (Sanders et al. 2016). Recent studies have shown that the local (intracrine) metabolism of steroids plays an as yet underappreciated role in functional fine-tuning in the endometrium (Gibson et al. 2020).

Another mechanism that was discussed was a secondary increase in the oestradiol concentration via the inhibition of sulfotransferase, leading to the development of uterine tumours (Wikoff et al. 2016). However, this is not plausible as an independent mechanism because the studies did not obtain the evidence necessary to support this by determining the oestradiol concentrations in the endometrium. Furthermore, none of the typical responses that are mediated by oestrogen receptors were observed such as a premature onset of puberty in female animals (NTP 2014) and no histological effects on the uterus (Cope et al. 2015; NTP 2014; Osimitz et al. 2016) or the oestrus cycle (Cope et al. 2015; NTP 2014) were found.

The tumours in the uterus of women are divided into 2 types: type I and type II carcinomas. While type I carcinomas are associated with hyperplasia and hyperoestrogenism, type II carcinomas are unrelated to oestrogen and characterized mainly by atrophy. Other important differences between type I and type II carcinomas are a loss of oestrogen and progesterone receptor expression as well as an increasing aggressiveness of the type II tumour. Endometrioid and mucinous tumours are examples of type I tumours and serous and clear cell carcinomas are examples of type II tumours. On a molecular level, the most frequent genetic alterations observed in type I carcinomas are PTEN (phosphatase and tensin homologue) inactivating mutations, microsatellite instability as well as CTNNB1 (encodes β-catenin) and KRAS mutations. The most frequent changes in type II carcinomas are TP53 mutations, the inactivation of CDKN2A (encodes p16) and CDH1 (encodes E-cadherin) as well as amplification of human epidermal growth factor receptor 2 (HER2, ERBB2) (Lax 2004, 2016). Atypical hyperplasia, the preneoplastic precursor, likewise involves the inactivation of PTEN, KRAS mutations and microsatellite instability (Lax 2004). The molecular genetics that contribute to the development of uterine carcinomas have thus been characterized in detail.

On a molecular level, uterine carcinomas induced by tetrabromobisphenol A are characterized by increased incidences of Tp53 mutations (incidence of mutations in tetrabromobisphenol A-induced adenocarcinomas: 15/22, 68%; controls: 2/10, 20%) and Ctnnb1 mutations (β-catenin = cadherin-associated protein, beta 1; incidence of mutations in tetrabromobisphenol A-induced adenocarcinomas: 3/22, 14%; controls: 0/10; 0%) as well as the mRNA overexpression of Her2 (Erbb2), Cdh1 (E-cadherin) and Ccnd1 (cyclin D1). Immunohistochemistry revealed that, like spontaneous adenocarcinomas, all substance-induced tumours were ERα-positive. Diffuse nuclear immunoreactivity for ERα was found in all cases. In 2 of 6 tetrabromobisphenol A-induced uterine carcinomas and 6 of 9 spontaneous tumours, positive immunoreactivity was found for PR. In some cases, the morphological and molecular features of tetrabromobisphenol A-induced adenocarcinomas resembled those of high-grade type I tumours (a high incidence of Tp53 mutations and an aggressive course as opposed to that of low-grade type I tumours) and type II tumours (overexpression of Her2) found in women. The authors, however, see more similarities with the high-grade type I carcinoma. While all carcinomas, irrespective of differentiation or induction by tetrabromobisphenol A, displayed nuclear expression of ERα, PR expression was reduced or lost in a majority of the substance-induced tumours and, to a lesser extent, in spontaneous tumours. The findings that tetrabromobisphenol A-induced tumours express ERα and tend to lose PR expression lead to the conclusion that these tumours remain oestrogen responsive, but tend to lose receptor functionality for progesterone. The high incidence of Tp53 mutations in substance-induced tumours in comparison with the incidence found in spontaneous tumours suggests that tetrabromobisphenol A-induced tumours are driven by a Tp53-dependent mechanism (Harvey et al. 2015).

As described above, tetrabromobisphenol A-induced uterine carcinomas in Wister Han rats and type I and type II carcinomas in women have similar molecular characteristics (Harvey et al. 2015). However, type I carcinomas are dependent on oestrogen and type II carcinomas are not (Lax 2004, 2016). This shows that not only effects that are caused by oestrogen, but also other oestrogen-independent effects are responsible for the development of tetrabromobisphenol A-induced uterine carcinomas. The NTP concurs with this assessment (NTP 2014).

A gene expression study in female Wistar Han rats found that tetrabromobisphenol A disrupts oestrogen homeostasis (Sanders et al. 2016). In the same strain, tetrabromobisphenol A downregulated genes that are associated with the immune response. This suggests that immunosuppressive effects are involved in the development of uterine tumours (Hall et al. 2017).

Another point that was discussed was that reproductive senescence begins at different time points in different rat strains and that oestrogen and progesterone concentrations vary. Wistar Han rats may therefore be more susceptible to uterine tumours than F344 rats (Lai et al. 2015). However, this was not investigated using tetrabromobisphenol A.

Like BDII/Han rats, female Wistar Han rats have a higher spontaneous incidence of atypical hyperplasia and adenocarcinomas of the uterus (Deerberg et al. 1981; Dixon et al. 2014; Weber 2017). The incidences of spontaneous adenocarcinomas in the uterus were 0.16% in F344 rats, 2.5% in Hsd:SD rats, 0.43% in Crl:CD(SD) rats, 2.72% in RccHan^TM^:WIST rats and 2.47% in Wistar Han rats (Weber 2017). A mean incidence of 2.47% (range: 0.89% to 14%) was determined for Wistar Han rats for the time period from 1997 to 2009 using data collected from 16 studies carried out in 4 different laboratories in the US, Canada and Europe (Charles River Laboratories 2011). As there is no long-term study available that investigated tetrabromobisphenol A in another rat strain, no conclusions can be drawn about possible differences between strains.

For many years, the NTP used Wistar Han rats for its carcinogenicity studies. However, the NTP stopped using this strain after several studies with perinatal exposure reported that the average litter sizes were smaller than expected, there was a high variability in the sex ratio of the litters and the incidence of pregnancy among time-mated rats was not in an acceptable range (NTP 2020). The Wistar Han [Crl:WI(Han)] strain, an outbred strain obtained from Charles River Laboratories (Raleigh, NC), was used for the carcinogenicity study with tetrabromobisphenol A (NTP 2014). The breeder has made a document concerning reproductive parameters available on its website that dates from 2009; this does not mention any abnormalities (Charles River Laboratories 2009). The cause of the reproductive issues is therefore unclear. They may be attributable to an increased production of hormones leading to secondary effects on organs that are sensitive to hormones. No data for the oestrus cycles of the animals are available from this time. Another study obtained Wistar Han rats from the breeder Charles River and bred the animals in-house in the laboratory. An evaluation of the oestrus cycles, beginning 2 weeks prior to the behavioural experiments (about postnatal day 76) and continuing up to the last time point of the behavioural experiments (about postnatal day 160), did not yield any unusual findings (Rock et al. 2019). As the animals were obtained for this study after the reproductive problems reported by the NTP occurred, no conclusion can be drawn whether the disruption of the oestrus cycle contributed to the reproductive problems.

### 2.9 Development of hepatoblastomas

In male B6C3F1/N mice, tetrabromobisphenol A doses of 250 mg/kg body weight and day and above did not lead to a dose-dependent increase in the incidence of hepatoblastomas or to eosinophilic foci in the liver (NTP 2014). Eosinophilic foci may be preneoplastic lesions, i.e. precursors of hepatocellular carcinomas (Thoolen et al. 2010).

Hepatoblastomas are less well-differentiated malignant tumours that may contain also liver cell carcinomas. Generally, however, these are not diagnosed separately. The exact pathogenesis of the hepatoblastomas is not known with certainty; it is assumed that they originate from hepatic stem cells, neoplastic hepatocytes or biliary epithelial cells (Thoolen et al. 2010). The pathogenesis of hepatoblastomas begins with foci that progress to hepatocellular adenomas and carcinomas and develop further into hepatoblastomas (Kim et al. 2005). Therefore, the eosinophilic foci can be regarded as an indirect precursor of tetrabromobisphenol A-induced hepatoblastomas.

Male B6C3F1 mice are more susceptible to substance-induced liver tumours than female mice (Maronpot et al. 1987). There is strong evidence that hepatocellular adenomas and carcinomas develop in rodents via CAR activation. This mechanism applies also to spontaneous tumours of this type (Elcombe et al. 2014; Lake et al. 2015; Peffer et al. 2018). Male B6C3F1 mice are more susceptible to developing hepatoblastomas after exposure to tetrabromobisphenol A than female mice (NTP 2014); this is consistent with a CAR/PXR-mediated mechanism of development.

The receptors CAR and PXR are often assessed together because there is extensive overlap in the functions of the 2 receptors and many substances activate both receptors (Peffer et al. 2018).

While the induction of CYP2B is more often mediated by CAR, PXR is considered the mediator of CYP3A regulation (Timsit and Negishi 2007). Tetrabromobisphenol A increased CAR expression and CYP2B1 induction in the liver of male Sprague Dawley rats given doses of 250 mg/kg body weight and day and above for 30 days (Choi et al. 2011), but did not induce CYP1A2, CYP2B1 or CYP3A1/3A3 in the liver of male and female Wistar rats given doses of up to 300 mg/kg body weight and day for 28 days (Germer et al. 2006). After the substance was administered to male and female F344/NTac rats in doses of 500 mg/kg body weight and day and above for 14 weeks, CYP2B activity in the liver was 4 to 23 times as high; the effects were stronger in the males than in the females (NTP 2014). There are no data for CAR expression in mice. The levels of CYP2B, CYP1A1, CYP1A2 and UGT (uridine 5ʹ-diphospho-glucuronosyltransferase) activity were not increased in B6C3F1/N mice given tetrabromobisphenol A doses of up to 1000 mg/kg body weight and day for 14 weeks (NTP 2014). As there are no signs of an increase in CYP2B activity, there is no evidence of CAR activation in mice. However, this study did not include the analysis of CYP2B protein and mRNA levels. The CYP3A activity was likewise not analysed; this could provide evidence of PXR activation in vivo (Timsit and Negishi 2007).

ToxCast data were analysed to identify interactions between tetrabromobisphenol A and PXR (IARC 2018; US EPA 2021 a). In general, it is plausible that the activation of CAR/PXR may be involved in the development of tetrabromobisphenol A-induced hepatoblastomas in male B6C3F1/N mice as well as their intermediates, hepatocellular adenomas and carcinomas.

In humans, nuclear transcription factors such as CAR and PXR likewise induce xenobiotic-metabolizing enzymes; however, the proliferative effects that are mediated by these receptors in the hepatocytes of rodents have not been found in humans (Elcombe et al. 2014; Lake et al. 2015; Li et al. 2015; Mackowiak et al. 2018; Ross et al. 2010). For this reason, several authors do not consider the hepatocellular tumours that are mediated by CAR and PXR in mice relevant to humans (Elcombe et al. 2014; Felter et al. 2018; Lake et al. 2015; Yamada et al. 2021). Other authors believe that the differences between humans and rodents are quantitative (Felter et al. 2018) and do not rule out human relevance (Braeuning et al. 2014). The Commission has concluded that the tumour is relevant to humans, but that humans are considerably less susceptible (Felter et al. 2018).

Using a microarray transcriptome analysis, various transcripts that are attributed to the interferon pathway (Irl-7, Mx1, Oas1, Isg15 (interferon-stimulated gene 15), Ddx60, Stat1 and Stat2) were induced in female Wistar-Han-IGS rats given gavage doses of tetrabromobisphenol A of 1000 mg/kg body weight and day for 13 weeks. In addition, the transcription of genes involved in the metabolism of xenobiotics or fatty acids (Scd2, Cyp2b6, Elovl6, Herc6, Fasn) was increased. The Ddx58 and Oas1 transcripts are known to be involved in the immune response. The transcript Isg15 is particularly important for the development of liver tumours and may play a role in the liver tumours found in B6C3F1/N mice (Dunnick et al. 2017).

### 2.10 Development of haemangiosarcomas

The incidence of haemangiosarcomas of all organs was significantly increased in male B6C3F1/N mice given tetrabromobisphenol A in doses of 500 mg/kg body weight and day and above; a statistically significant trend is evident. Tumour incidences were not increased at 250 mg/kg body weight and day (NTP 2014).

Haemangiosarcomas are aggressive, malignant tumours of the endothelial cells. They are believed to arise from a number of different types of endothelial cells such as hepatic sinusoidal cells, venous, arterial and capillary endothelia and from bone marrow-derived stem cells. As mature endothelial cells continue to proliferate in adulthood, there is a risk of neoplastic transformation. Haemangiosarcomas can arise in any organ (Cohen et al. 2009).

In CD-1 and B6C3F1 mice, PPARγ and dual PPARα/γ agonists increased the incidence of vascular tumours. For most of the investigated substances, the haemangiosarcomas induced in multiple tissues are consistent with the sites of spontaneous tumour formation (Hardisty et al. 2007). The PPARγ agonist troglitazone, for example, induced haemangiomas and haemangiosarcomas in mice. This is attributed to a direct mitogenic effect in the endothelial cells in brown and white adipose tissue and in the liver (Kakiuchi-Kiyota et al. 2009, 2011). As tetrabromobisphenol A has been shown to be a potent PPARγ agonist in humans (Honkisz and Wójtowicz 2015 b; Riu et al. 2011 a, b) or partial PPARγ agonist in mice (Watt and Schlezinger 2015), PPARγ is assumed to play at least some role in the development of haemangiosarcomas. An analysis of the ToxCast data identified interactions between tetrabromobisphenol A and PPARγ (IARC 2018; US EPA 2021 a). According to the ToxCast database, there is an adverse outcome pathway (AOP number 163) that describes how the activation of PPARγ can lead to the development of sarcomas in rats, mice and hamsters (US EPA 2021 a). Proliferative effects induced by tetrabromobisphenol A in the endothelial cells of mice have not been investigated. The basal proliferation rates of endothelial cells in the liver of male B6C3F1 mice were about 3 to 5 times those determined in male F344 rats and human males. The rates were likewise higher than those determined in the females of the different species. The increased incidences of spontaneous and PPARγ agonist-induced haemangiosarcomas in mice are probably attributable to the increased basal proliferation rate of endothelial cells in B6C3F1 mice in comparison with the rates determined in F344 rats and humans (Ohnishi et al. 2007). There is no evidence that the effect is specific to a particular species; the Commission thus assumes that this type of tumour is relevant to humans.

### 2.11 Conclusions: mechanism of action

Tetrabromobisphenol A interacts with nuclear receptors. The substance thereby changes the function of the thyroid hormone receptor directly and indirectly (Butt et al. 2011; Freitas et al. 2011; Guyot et al. 2014; Hamers et al. 2006; Hofmann et al. 2009; Jugan et al. 2007; Kitamura et al. 2002, 2005 a, b; Lévy-Bimbot et al. 2012; Meerts et al. 2000; Oka et al. 2013; Sun et al. 2009; Terasaki et al. 2011; Yamada-Okabe et al. 2005) and has been shown to activate PPARγ (Chappell et al. 2018; Honkisz and Wójtowicz 2015 b; Riu et al. 2011 a, b; Watt and Schlezinger 2015). The oestrogen receptors ERα und ERβ were not activated or only very weakly (Bermudez et al. 2010; Kitamura et al. 2002, 2005 b; Li et al. 2010; Olsen et al. 2003; Samuelsen et al. 2001). An antagonistic effect is not known at these oestrogen receptors (Judson et al. 2015). In addition, a direct and indirect binding affinity to the non-nuclear G-protein-coupled oestrogen receptor (GPER) was observed (Cao et al. 2017; Chappell et al. 2018; Hoffmann et al. 2017 b). Mechanistically, however, plausible evidence of the involvement of this receptor in the induction of uterine carcinomas by tetrabromobisphenol A in Wistar Han rats has not been found.

Tetrabromobisphenol A was shown to be a potent inhibitor of the sulfotransferase SULT1E1 that metabolizes oestradiol (Gosavi et al. 2013; Hamers et al. 2006; Harju et al. 2007; Kester et al. 2002; Wikoff et al. 2016); this could lead to an increased bioavailability of oestradiol (Borghoff et al. 2016; Wikoff et al. 2016). However, although the effect is theoretically plausible, there is no evidence to support this. For this reason, there is no NOAEL (no observed adverse effect level) for the (postulated) increase in the oestrogen concentration. A gene expression study in female Wistar Han rats found evidence suggesting impaired oestrogen homeostasis and immunosuppressive effects caused by tetrabromobisphenol A (Hall et al. 2017; Sanders et al. 2016).

In addition, the substance leads to mitochondrial dysfunction (Choi et al. 2017; Nakagawa et al. 2007; Ogunbayo et al. 2008; Suh et al. 2017; Ziemińska et al. 2012, 2017), oxidative stress (in vitro: Al-Mousa and Michelangeli 2012; Choi et al. 2017; Nakagawa et al. 2007; Reistad et al. 2005, 2007; Suh et al. 2017; Ziemińska et al. 2012, 2017; in vivo: Chignell et al. 2008; Choi et al. 2011; Kang et al. 2009; Szymańska et al. 2000) and to immunosuppressive effects (in vitro: Almughamsi and Whalen 2016; Anisuzzaman and Whalen 2016; Choi et al. 2017; Hurd and Whalen 2011; Kibakaya et al. 2009; Pullen et al. 2003; Yasmin and Whalen 2018; in vivo: Hall et al. 2017; Watanabe et al. 2010, 2017). Proinflammatory effects in vitro (Arita et al. 2018; Han et al. 2009; Koike et al. 2013; Park et al. 2014; Suh et al. 2017) and the disruption of signal cascades and of complex gene regulation systems were likewise observed in vitro and in vivo (Cato et al. 2014; Hall et al. 2017; Honkisz and Wójtowicz 2015 b; Lenart et al. 2017; Lévy-Bimbot et al. 2012; Reistad et al. 2005, 2007; Sanders et al. 2016; Strack et al. 2007).

Non-genotoxic mechanisms are responsible for tumour development. The formation of uterine tumours in Wistar Han rats is attributed to oestrogenic and non-oestrogenic effects. A gene expression study in female Wistar Han rats reported that the disruption of oestrogen homeostasis by tetrabromobisphenol A was not mediated by an agonistic effect on the oestrogen receptors ERα and ERβ (Sanders et al. 2016). The oestrogenic effects lead to proliferation in the endometrium (NTP 2014). One example of a non-oestrogenic effect is immunosuppression. The development of hepatoblastomas in male B6C3F1/N mice is consistent with the CAR/PXR induction mechanism that is known to occur in this mouse strain. The eosinophilic foci are to be regarded as an indirect precursor that first progresses to hepatocellular carcinomas and then to hepatoblastomas. The Commission assumes that this tumour is relevant to humans, but that humans are considerably less susceptible (Felter et al. 2018). An association has been found between the higher basal proliferation rate of endothelial cells in B6C3F1/N mice in comparison with the rates found in F344 rats and humans, PPARγ-agonistic effects and the development of haemangiosarcomas in male B6C3F1/N mice. There is no evidence that the effect is species-specific. The Commission therefore assumes that the tumour is relevant to humans.

## 3 Toxicokinetics and Metabolism

### 3.1 Absorption, distribution, elimination

#### 3.1.1 Humans

In a toxicokinetics study, 5 test persons (3 men, 2 women) were each given a single oral dose of tetrabromobisphenol A of 0.1 mg/kg body weight in the form of a gel capsule. Urine samples were taken immediately after administration and after 3 to 178 hours. Blood samples were likewise taken immediately after administration and after 1 to 178 hours. At all time points, the concentration of unchanged parent substance was below the limit of detection both in the plasma and in the urine (3 to 4 nmol/l for tetrabromobisphenol A, its glucuronide and sulfate). The maximum concentration of tetrabromobisphenol A-glucuronide in the plasma was reached 2 to 6 hours after administration and was 16 nmol/l. The glucuronide was present in the urine in quantifiable concentrations at all time points. Total excretion peaked at 4 nmol after 63 hours. Only a small percentage of less than 0.1% of the dose was recovered in the urine in the form of the glucuronide. It was not possible to detect a specific pattern of excretion kinetics in the urine. The AUCs (areas under the curve) of the glucuronide were 0.79 nmol/ml × hour in the plasma and 0.17 nmol × hour in the urine. Four to 6 hours after administration, tetrabromobisphenol A-sulfate was detected in the plasma of 2 test persons in a concentration of 20 µmol/l. The sulfate concentration was below the level of detection in all urine samples. Therefore, it was not possible to calculate an AUC for the sulfate (Schauer et al. 2006).

In a study that investigated 4 employees with occupational exposure at a disassembly facility for electronic devices in Sweden, tetrabromobisphenol A concentrations of 2 to 7 pmol/g blood lipids were determined in serum samples collected prior to the summer holidays in 1997 and 1998. The summer break lasted 25 to 31 days and determinations were taken several times during this time period. A half-life of 2.2 days was obtained for tetrabromobisphenol A in the serum (Hagmar et al. 2000).

#### 3.1.2 Rat

##### 3.1.2.1 Inhalation

There are no toxicokinetics studies with inhalation exposure available.

##### 3.1.2.2 Oral administration

Six male Sprague Dawley rats were given a single gavage dose of tetrabromobisphenol A of 300 mg/kg body weight (vehicle: corn oil). After 3 hours, the tetrabromobisphenol A concentration in the plasma reached its highest level at 103 µmol/l; the concentration then decreased with a half-life of 13 hours. The initial half-life was about 4 hours (estimated from a figure). The highest concentration of the tetrabromobisphenol A-glucuronide in plasma of 25 µmol/l was likewise determined after 3 hours; the tetrabromobisphenol A-sulfate reached its peak at 694 µmol/l after 6 hours. The AUCs in plasma were 1028 (tetrabromobisphenol A), 161 (glucuronide) and 9251 (sulfate) nmol/ml × hour and the AUCs in urine 21 (glucuronide) and 12 482 (sulfate) nmol × hour. The tetrabromobisphenol A concentration in the urine was below the limit of detection (Schauer et al. 2006).

In a toxicokinetics study, groups of 4 male F344 rats were administered ^14^C-labelled tetrabromobisphenol A in single gavage doses of 2, 20 and 200 mg/kg body weight or as a single intravenous injection of 20 mg/kg body weight. At all doses, more than 90% of the administered radioactivity had been excreted with the faeces after 72 hours. The largest fraction was excreted during the first 24 hours. In the high dose group, 0.2% to 0.9% of the ^14^C was found in the tissues after 72 hours. About 50% of the oral dose of 20 mg/kg body weight was recovered in the bile within 2 hours. Tetrabromobisphenol A was readily absorbed in the intestines, but oral bioavailability remained below 2%. After 20 mg/kg body weight and day was administered orally for 5 or 10 days, cumulative excretion with the faeces at the end of exposure was 85.1% and 97.9%, respectively. In all studies, the amount of radioactivity recovered in the urine was below 2%. Accumulation in the tissues was not detected after repeated exposure. The majority (75%) of the substance was likewise excreted with the faeces after intravenous injection. The amount of radioactivity in the blood decreased rapidly after intravenous injection and a 2-compartment model could describe the elimination kinetics. The terminal elimination half-life was 82 minutes and the AUC was 1440 μg/ml × min. Overall, after largely complete absorption, there is an extensive first-pass effect in the liver and low systemic bioavailability (Kuester et al. 2007).

Male Sprague Dawley rats, 8 with bile duct cannulas, 10 without, were given a single oral dose of ^14^C-labelled tetrabromobisphenol A of 2 mg/kg body weight. In the conventional rats, 91.7% of the substance was excreted with the faeces and only 0.3% with the urine. In the animals with bile duct cannulation, 71.3% was excreted with the bile, 26.7% with the faeces and 0.7% with the urine. In the conventional rats, 2% of the ^14^C activity was detected in the tissues. In the bile duct-cannulated animals, the level of activity was less than 1%. The majority of the ^14^C activity was found in the small and large intestines, irrespective of whether the cannulation was placed into the bile duct. More than 95% of the extractable ^14^C in the faeces was identified as unmetabolized parent substance (Hakk et al. 2000). Oral absorption was calculated to be about 72% from the data for the bile duct-cannulated animals.

Groups of 4 female Crl-WI(Han) rats were given ^14^C-labelled tetrabromobisphenol A in single gavage doses of 25, 250 or 1000 mg/kg body weight or by intravenous injection (in a ratio of ethanol : water : cremophore EL of 1:3:1) of 25 mg/kg body weight. After administration of the oral dose of 250 mg/kg body weight, the compound was rapidly absorbed into systemic circulation, reaching its maximum concentration in plasma after 1.5 hours. This was followed by a longer terminal phase. The highest amounts of radioactivity were detected in the liver and in the pancreas. After intravenous injection, the tetrabromobisphenol A concentration in the plasma decreased rapidly, followed by a long elimination phase. After oral administration, tetrabromobisphenol A was excreted mainly with the faeces. After administration of the 3 oral doses, between 94.3% and 98.8% of the applied radioactivity was recovered in the faeces, 0.2% to 2% in the urine, less than 0.1% in the tissues and less than 0.01% in the uterus. The elimination pathways were saturated at 1000 mg/kg body weight, leading to delayed excretion. The elimination half-life of tetrabromobisphenol A in the plasma was about 290 minutes after oral administration and 133 minutes after intravenous injection. The bioavailability after oral administration was below 5% (Knudsen et al. 2014) and thus similar to the value determined in male F344 rats (Kuester et al. 2007).

Groups of 5 male Sprague Dawley rats were given tetrabromobisphenol A by gavage, either as a single dose or as repeated doses for 14 days at levels of 200, 500 or 1000 mg/kg body weight and day. After the administration of single doses of tetrabromobisphenol A of 200, 500 and 1000 mg/kg body weight, maximum concentrations in the blood of 12.5, 18.65 and 31.27 µg/ml, respectively, were reached within 5 hours. The terminal half-lives were between 7.2 and 9.5 hours. A 14-day study did not detect any accumulation in the organism (Kang et al. 2009).

Groups of 4 to 6 female Wistar Han rats were given gavage doses of tetrabromobisphenol A of 0, 50, 100, 250, 500 and 1000 mg/kg body weight and day in corn oil on 28 consecutive days. The concentrations of tetrabromobisphenol A, its glucuronide and its sulfate were determined in the plasma (4 and 8 hours after administration on days 7, 14 and 28), in the liver and in the uterus (after 4 and 8 hours on day 28). The concentrations of the parent substance and of the glucuronide and sulfate conjugates increased with the dose in the plasma, liver and uterus. The concentrations of tetrabromobisphenol A-glucuronide and tetrabromobisphenol A-sulfate in the plasma were higher in the animals after 28 days than after 7 and 14 days; this shows an increase in the systemic circulation of these 2 conjugates. At doses of 500 and 1000 mg/kg body weight and day, a higher concentration of sulfate than of glucuronide was found in the liver after 28 days; the opposite was true for the plasma and uterus. In all 3 tissues, the ratio tetrabromobisphenol A-sulfate : tetrabromobisphenol A-glucuronide decreased with the dose; this suggests a limited capacity for sulfation (see also Section 2.8; Borghoff et al. 2016). Sulfates are evaluated by assessing the capacity for sulfation as a whole and not only the fraction catalysed by oestrogen-specific sulfotransferases. The capacity for sulfation remains even at higher doses, as shown by the increasing fraction of sulfoconjugates. However, these findings cannot be used to determine whether oestrogen-specific sulfotransferase is saturated. Metabolism shifts from sulfation towards glucuronidation with the increasing dose of tetrabromobisphenol A.

Sulfation is a “high affinity – low capacity” system, while glucuronosyltransferases are a “low affinity – high capacity” system. Sulfation reactions underlie a rapid initial metabolic rate, particularly at low concentrations. However, the reaction rate rapidly declines because of the limited availability of the co-factor 3′-phosphoadenosine-5′-phosphosulfate (PAPS). Conversely, humans endogenously produce the co-factor uridine-5′-diphospho-α-D-glucuronic acid in large amounts of up to 5 g a day. This cofactor can be reproduced rapidly (Klaassen and Boles 1997; Testa and Krämer 2008).

The evidence suggests that tetrabromobisphenol A undergoes enterohepatic circulation in rats (Hakk et al. 2000; Knudsen et al. 2014).

##### 3.1.2.3 Dermal application

^14^C-labelled tetrabromobisphenol A in acetone was applied non-occlusively to 1 cm^2^ (0.054 or 0.54 mg) of shaved skin on the backs of 4 female Wistar Han rats in concentrations of either 100 nmol/cm^2^ (0.25 mg/kg body weight, 24 hours) or 1000 nmol/cm^2^ (2.5 mg/kg body weight, 24 or 72 hours). In the low dose group, 13.6% of the substance was recovered at the application site after 24 hours, while 7.7% entered systemic circulation. In the high dose group, the sum of both values was about 10%; however, the sum increased to about 70% in the animals with 72-hour application. After application for 24 hours, 5.5% and 7.3%, respectively, were excreted with the urine and faeces (Knudsen et al. 2015). On the basis of these data, amounts of 0.3 and 2.4 mg per 2000 cm^2^ would be absorbed after exposure for 1 hour.

**Fig. 1:**
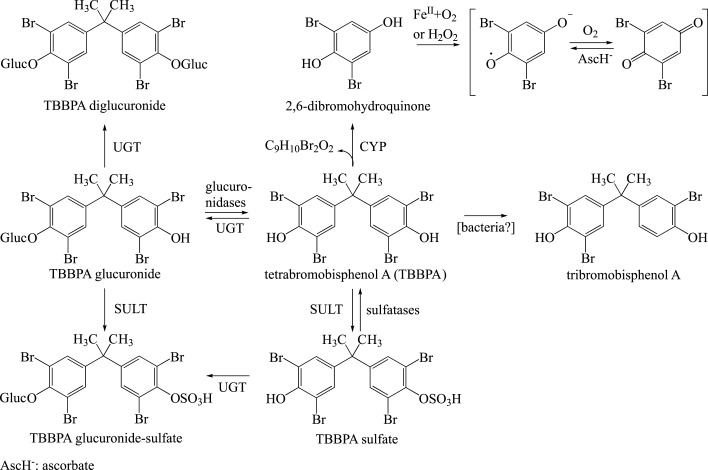
Metabolism of tetrabromobisphenol A in rats (NTP 2014), modified

Tetrabromobisphenol A was applied semi-occlusively to 36 cm^2^ of shaved skin (10.6 to 288 mg) on the backs of groups of 6 male Wistar rats in doses of 0, 20, 60, 200 and 600 mg/kg body weight and day (particle sizes 0.1–100 µm) for 6 hours a day on 90 days. The substance was moistened with physiological saline. The serum concentrations of tetrabromobisphenol A ranged from 19.04 to 427.20 g/g lipids (the unit µg/g lipids was used in Figure 1 of the publication. This seems more plausible in light of the administered dose). The percentages of recovered tetrabromobisphenol A were between 0.002% ± 0.002% and 0.013% ± 0.008% in the serum, between 0.004% ± 0.002% and 0.072% ± 0.027% in the urine and between 3.30% ± 0.61% and 11.13% ± 3.16% in the faeces. Dermal absorption was between 3% and 11% of the total administered dose (Yu et al. 2016). On the basis of these data, amounts of 10.9 to 88.4 mg per 2000 cm^2^ would be absorbed after exposure for 1 hour.

Tetrabromobisphenol A was applied semi-occlusively to 36 cm^2^ of shaved skin (5 to 173 mg) on the backs of groups of 6 male and 6 female Wistar rats in doses of 20, 60, 200 and 600 mg/kg body weight (particle sizes 0.1–100 µm) for an exposure period of 6 hours. The substance was moistened with physiological saline. The skin adhesion coefficients of the male and female animals were 0.12 to 3.25 mg/cm^2^ and 0.1 to 2.56 mg/cm^2^, respectively. The adhesion rate was 70.92%. The diffusion constant was 1.4 × 10^–4^ cm^2^/h and the permeation coefficient (Kp) was 1.26 × 10^–5^ cm/h. Male rats had higher tetrabromobisphenol A concentrations in the blood, urine and faeces than female rats. The amount of substance recovered in the urine, faeces and serum within 24 hours ranged from 1.37 to 39.09 mg in male rats and 0.73 to 33 mg in female rats (Yu et al. 2017). On the basis of these data, amounts of 6.8 to 361.9 mg per 2000 cm^2^ would be absorbed after exposure for 1 hour.

##### 3.1.2.4 Intraperitoneal injection

Groups of 4 female IMP:WIST rats were given a single intraperitoneal injection of ^14^C-labelled tetrabromobisphenol A in doses of 250 or 1000 mg/kg body weight. The radioactivity in the erythrocytes was 10 times that in the plasma 72 hours after administration. The highest ^14^C values were found in the adipose tissue, followed by the liver, sciatic nerve, muscles and adrenal glands (only certain organs were examined, no other details). After 72 hours, the total amount excreted with the faeces was 51% to 65% of the applied dose, while only 0.3% was excreted with the urine. About 20% of the applied dose remained in the animals (Szymańska et al. 2001).

##### 3.1.2.5 Pregnant animals

Pregnant Wistar Han IGS rats were administered a single gavage dose of ^14^C-labelled tetrabromobisphenol A of 25 mg/kg body weight on gestation day 20. The animals were examined after 0.5, 1, 2, 4, 8 and 24 hours. The radioactivity in the blood varied very little in the time period between 0.5 and 8 hours and was below the limit of detection after 24 hours (3 times the background level) with an absorption half-life of 16 minutes and an elimination half-life of 17 hours. Both tetrabromobisphenol A and its metabolites were detected in the plasma after 30 minutes, while at all other time points only the metabolites were found. The maximum concentration of about 2 nmol equivalents/g (estimated based on a figure) was determined in the total foetus 2 hours after dose administration. At all other time points, the fraction of the applied radioactivity detected in the foetuses was below 1% (Knudsen et al. 2018).

Pregnant rats (no other details) given gavage doses of ^14^C-labelled tetrabromobisphenol A of 190 μg/kg body weight and day from gestation days 16 to 19 were examined on gestation day 20. The radioactivity determined in the animals was less than 0.5% of the dose, whereby the highest levels of radioactivity were found in the intestines and in the liver. In the foetuses, the fraction was less than 0.01% of the applied dose, which suggests low transplacental transfer (EFSA 2011).

#### 3.1.3 In vitro

^14^C-labelled tetrabromobisphenol A was applied to samples of split-thickness skin from humans and rats (application area: 0.64 cm^2^) in a concentration of 100 nmol/cm^2^. Absorption was determined with flow-through cells. Hanks’ medium with foetal calf serum was used as the receptor fluid. The radioactivity was determined in 6-hour intervals up to 24 hours after administration. On average, 3.4% and 0.2%, respectively, of the applied dose were detected in the human skin and its receptor fluid, whereas 9.3% and 3.5%, respectively, were found in the rat skin and its receptor fluid. On the basis of the data from in vitro and in vivo studies in rats (see above), humans are estimated to absorb up to 6% of the substance through the skin (Knudsen et al. 2015).

### 3.2 Metabolism

#### 3.2.1 Humans

Tetrabromobisphenol A-glucuronide and sulfate were identified as metabolites in the blood and urine of humans exposed by an oral route of administration (Schauer et al. 2006).

#### 3.2.2 Rat

Figure 1 depicts the metabolism of tetrabromobisphenol A in rats.

The 2 major metabolites found in the blood of rats were the glucuronide and sulfate conjugates of tetrabromobisphenol A. Other metabolites were the diglucuronide, a mixed glucuronide–sulfate conjugate of tetrabromobisphenol A and, in low concentrations, tribromobisphenol A and its glucuronide (Schauer et al. 2006). The conversion to glucuronides and sulfates is catalysed by glucuronosyltransferases and sulfotransferases (Hakk et al. 2000; Knudsen et al. 2014). Tetrabromobisphenol A is a poor substrate for the aryl sulfotransferase system because of steric hindrance by the ortho position of bromine. As a result, the glucuronosyltransferase system is favoured (Hakk et al. 2000).

An electron paramagnetic resonance (EPR) test detected radicals in the bile of male Sprague Dawley rats given a single intraperitoneal injection of tetrabromobisphenol A dissolved in dimethyl sulfoxide (DMSO) in doses of 100 or 600 mg/kg body weight. The spin trap reagent, α-(4-pyridyl-1-oxide)-*N*-*t*-butylnitrone (POBN) dissolved in water, was administered by intraperitoneal injection. The radical was identified as POBN/•CH_3_. Also present in the bile was the 2,6-dibromobenzosemiquinone radical, which made up 25% of the total EPR intensity. This radical is a product of 2,6-dibromohydroquinone, another metabolite of tetrabromobisphenol A. The reaction of the 2,6-dibromobenzosemiquinone radical with oxygen generates superoxide, from which hydrogen peroxide can form by dismutation. The hydroxyl radical generated via the Fenton reaction from hydrogen peroxide reacts in vivo with DMSO to give the methyl radical which is trapped by POBN (Chignell et al. 2008). This system is very artificial. The methyl radical that is captured in the spin trap is generated from the DMSO formed via H_2_O_2_ in the redox cycling process.

Monoglucuronide and monosulfate conjugates formed from tetrabromobisphenol A also in isolated rat hepatocytes after the addition of a metabolic activation system (Nakagawa et al. 2007; Zalko et al. 2006).

The main reaction pathway in rat microsomes is oxidative cleavage of the tetrabromobisphenol A molecule, thereby forming hydroxylated dibromo-phenol, hydroxylated dibromo-isopropyl-phenol and glutathione-conjugated dibromo-isopropyl-phenol. The 2 main metabolites are 2 molecules of lower polarity than the parent compound that were characterized as a hexa-brominated compound with 3 aromatic rings and a hepta-brominated dimer-like compound and identified as 2,6-dibromo-4-(2′,6′-dibromo-1′-hydroxycumyl)phenoxy-3′′,5′′-dibromo-4′′-hydroxybenzene (M7a) and 2,6-dibromo-4-(2′,6′-dibromo-1′-hydroxycumyl)phenoxy-2′′-hydroxy-3′′-bromo-5′′-(2′,6′-dibromo-1′-hydroxycumyl)benzene (M7b). As they form only in NADPH-generating systems, CYP-dependent oxidation of tetrabromobisphenol A must be the first step. The structure of the metabolite M7b suggests nucleophilic substitution of a bromine of one molecule of tetrabromobisphenol A by a second molecule of tetrabromobisphenol A. One explanation for the formation of M7a is that tetrabromobisphenol A undergoes CYP-dependent hydroxylation, forming a reactive radical intermediate. Two molecules of this hydroxylated intermediate could then disproportionate with concomitant cleavage and dehydration, giving rise to tetrabromobisphenol A and hydroxydibromophenol. These combine to produce the metabolite M7a by dehydration. On the other hand, disproportionation gives rise to an intermediate radical of dibromo-isopropyl-phenol. This is recovered in incubation supernatants as a glutathione conjugate or as hydroxylated metabolites. Qualitatively, tetrabromobisphenol A follows similar metabolic pathways in the liver microsomes of humans and rats. Tetrabromobisphenol A-glucuronide formed more rapidly after the addition of a human metabolic activation system than after the addition of one from rats (Zalko et al. 2006). Hydroxylated 2,6-dibromo-4-phenol (M1) is the precursor of the 2,6-dibromobenzo-semiquinone radical (see above Chignell et al. 2008). In vitro, this makes up less than 1% of the total radioactivity (Zalko et al. 2006). In vivo evidence of the oxidative metabolites described in the study has yet to be obtained; there is only indirect evidence of hydroxylated 2,6-dibromo-4-phenol.

#### 3.2.3 Sulfotransferases

As tetrabromobisphenol A is a potent inhibitor of the SULT1E1 sulfotransferase that metabolizes oestradiol (Gosavi et al. 2013; Hamers et al. 2006; Harju et al. 2007; Kester et al. 2002; Wikoff et al. 2016), sulfotransferases are described briefly below.

Sulfotransferases transfer a sulfo group from PAPS onto the acceptor group of their substrates. Chemically, this is a sulfonation. A sulfate ester (R-OSO_3_^–^) forms if the acceptor is a hydroxyl group, hence the term sulfation is commonly used.

The sulfation of tetrabromobisphenol A is catalysed via the enzymes SULT1A1 and SULT1E1 (Hamers et al. 2006; Kester et al. 2002).

The isoenzymes of the SULT1A subfamily are also called phenol SULTs because they catalyse the sulfation of phenolic molecules. SULT1E isoenzymes are responsible for catalysing the sulfation of the 3α-hydroxy group of endogenous substances (for example: oestrone and β-oestradiol) as well as of xenobiotics (for example: 17α-ethinyloestradiol), generally also with high affinity (Blanchard et al. 2004).

Many tissues in humans express SULT1A1 and SULT1E1. SULT1A1 is expressed at high levels in the liver, but also in the brain, endometrium, intestines, kidneys and lungs. SULT1E1 is found particularly in tissues sensitive to steroid hormones such as the endometrium, testes, breast, placenta, vagina and adrenal glands, but also in the liver and brain (Blanchard et al. 2004; Gamage et al. 2006; Glatt 2000; Riches et al. 2009). Different SULT1A1 polymorphisms with different types of activity have been described (Tremmel et al. 2017). In humans and in other species of mammal, SULT1E1 activity is suppressed during the proliferative phase (follicular phase) of the cycle when oestrogen is active and high during the luteal phase of the cycle when the effects of oestrogen and cell proliferation have to be lower to support secretory processes (Demyan et al. 1992; Falany et al. 1998). It is highly probable that the expression of SULT1E1 in the endometrium of women is regulated by progesterone, a hormone that is produced by the ovaries (Falany et al. 1998). The Michaelis-Menten constant (K_m_ value) for oestradiol and oestrone is 5 nM for human SULT1E1 in the uterus (Falany et al. 1998). Human SULT1E1 has an active and an allosteric site. Oestradiol can bind to both sites (Sun and Leyh 2010; Zhang et al. 1998). For this reason, oestradiol is able to act also as a partial inhibitor (inhibition constant: K_i_ 80 nM; Zhang et al. 1998; IC_50_: 3.8 to 7.1 nM; Kester et al. 2002).

In rats, sex-specific differences have been found in the metabolic processes catalysed by sulfotransferases (Alnouti and Klaassen 2011). SULT1A1 predominates in male rats and is expressed in the liver, brain, lungs, heart, intestines, kidneys, adrenal glands, testes and spleen (Dunn and Klaassen 1998). SULT1A1 expression in the liver increases in male and female rats with age, reaching its peak at an age of 30 days. This is followed by a decline. SULT1A1 is expressed in male rats at levels that are up to twice as high as those found in females (Liu and Klaassen 1996 b). In female rats, SULT1E1 is expressed in the liver and various reproductive organs such as the uterus, placenta and mammary glands (Demyan et al. 1992). While SULT1E1 is expressed at low levels in the liver of female rats throughout their lifetime, expression begins to increase in male rats with puberty, reaching levels that are 10 times as high as those found in females. While the mRNA of the SULT1E2 isoform was detected in male rats beginning at an age of 30 days and its level increased with age, the mRNA of this isoform was not found in female rats (Klaassen et al. 1998; Liu and Klaassen 1996 a).

SULT1A1 is expressed in many tissues also in mice. The highest levels were found in the intestines, liver and lungs. Sex-specific differences have been observed. SULT1A1 was suppressed by androgen in the liver of male mice. SULT1E1 is expressed at high levels in the placenta and uterus of female mice and in the gonads of male mice, but expressed at low levels in all other tissues (Alnouti and Klaassen 2006, 2011; Böhmdorfer et al. 2017). At an age of 45 days, the hepatic expression of SULT1A1 in female mice was more than 100 times the level determined in male mice (Alnouti and Klaassen 2006).

Studies comparing the sulfation of tetrabromobisphenol A in different species are not available.

## 4 Effects in Humans

### 4.1 Single exposures

There are no data available.

### 4.2 Repeated exposure

A biomonitoring study in adolescents (Kiciński et al. 2012) is not described or included in this evaluation because it is not relevant to the workplace.

### 4.3 Local effects on skin and mucous membranes

There are no data available.

### 4.4 Allergenic effects

A Landsteiner-Draize test was carried out with repeated application of 3 to 5 mg tetrabromobisphenol A. The substance was mixed to a paste with water (concentration 50% to 70%) and applied occlusively to the upper arm for a total of ten applications, each lasting 48 to 72 hours. Tetrabromobisphenol A produced a reaction in 1 of 54 volunteers directly after 48-hour occlusive challenge treatment using the same amount; this reaction subsided after 72 hours. It is regarded as an irritant reaction (International Research and Development Corporation 1978 c, pp.153–165).

### 4.5 Reproductive and developmental toxicity

The mean concentration of tetrabromobisphenol A in the serum of 26 infants with congenital hypothyroidism was 83.4 ng/g blood lipids and thus considerably higher than the value of 8.89 ng/g blood lipids determined in the serum of their mothers. A weak correlation was found between the concentration of tetrabromobisphenol A and thyroid hormones in mother–infant pairs with infants with congenital hypothyroidism (correlation coefficient for free T4: 0.430). A statistically significant difference between the tetrabromobisphenol A concentrations in the serum of infants with congenital hypothyroidism and those of the control group without a thyroid disorder was not observed (Kim and Oh 2014).

### 4.6 Genotoxicity

There are no data available.

### 4.7 Carcinogenicity

There are no data available.

## 5 Animal Experiments and in vitro Studies

### 5.1 Acute toxicity

#### 5.1.1 Inhalation

Groups of 5 male and 5 female Wistar rats, NMDI mice and guinea pigs (strain not specified) were exposed whole-body to a tetrabromobisphenol A concentration of 0.5 mg/m^3^ for 8 hours. The aerosol was generated by a Draeger/Luebeck device. A continuous flow of air was maintained for 8 hours. Toxic effects were not observed during exposure or during the 2-day observation period. No unusual findings were observed at necropsy at the end of the observation period (no other data; International Bio-Research Inc. 1967 b, pp. 205–207).

Ten male Dublin albino rats were exposed whole-body to tetrabromobisphenol A vapour in a concentration of 1237 ml/m^3^ for 1 hour. The concentration was calculated and not determined by analysis. No animal died during the 14-day observation period. The body weights remained within the normal range for this strain (Hill Top Research Inc. 1966, pp. 10–20).

#### 5.1.2 Oral administration

In a test carried out with rats according to OECD Test Guideline 401, the oral LD_50_ was above 2000 mg/kg body weight (no other details; MHLW 2019 a, e).

In a study carried out according to valid test guidelines, 5 male and 5 female Sprague Dawley rats were given a single dose of tetrabromobisphenol A of 5000 mg/kg body weight in 0.25% aqueous methyl cellulose. No animal died during the 14-day observation period. In addition, necropsy did not reveal any toxic effects or unusual findings. Therefore, the oral LD_50_ was above 5000 mg/kg body weight (EU 2006).

Ten Holtzman rats were given a single gavage dose of tetrabromobisphenol A of 50 mg/kg body weight in distilled water. None of the animals died during the 48-hour observation period (no other details; Leberco Laboratories 1958).

Groups of 5 male Dublin albino rats were given a single gavage dose of tetrabromobisphenol A of 100, 215, 464, 1000, 2150, 4640 or 10 000 mg/kg body weight in corn oil. Two animals of the high dose group died during the 14-day observation period. No effects on body weight gains were observed. The oral LD_50_ was thus about 10 000 mg/kg body weight (no other details; Hill Top Research Inc. 1966, pp. 10–20).

Five male and 5 female Wistar rats were given a single gavage dose of tetrabromobisphenol A of 50 000 mg/kg body weight in 0.25% methyl cellulose. All animals showed slight to moderate apathy and 3 animals died within 5 hours of receiving the dose. At necropsy, no findings were observed in the deceased animals or in the animals that survived and were examined 14 days later (International Bio-Research Inc. 1967 c, pp. 211–213).

In a range-finding study that was not described in detail, 2 female rats per dose group (strain not specified) were given a single gavage dose of tetrabromobisphenol A of 250, 500, 1000, 2000 or 4000 mg/kg body weight in corn oil. None of the animals died. At necropsy, slight liver damage was found in the rats given 1000 mg/kg body weight and moderate liver and kidney damage in those given 2000 and 4000 mg/kg body weight. No further data were provided. Therefore, the oral LD_50_ was above 4000 mg/kg body weight (no other details; EU 2006). Other studies did not report findings of these types of liver effects at the same or higher doses. Therefore, the results are not reliable.

Oral LD_50_ values of above 2000 mg/kg body weight and of 3200 mg/kg body weight were found in rats and mice, respectively (no other details; WHO 1995).

Groups of 5 male and 5 female CD1 mice were given gavage doses of tetrabromobisphenol A of 1000, 1585, 2512, 3980, 6308 and 10 000 mg/kg body weight and observed for 14 days. The oral LD_50_ was above 10 000 mg/kg body weight (International Research and Development Corporation 1978 a, pp. 133–141).

##### 5.1.2.1 Neurotoxicity

Groups of 8 male NMRI mice (10 days old) were given a single gavage dose of tetrabromobisphenol A of 0, 0.75 or 11.5 mg/kg body weight in a lipid emulsion. Several behavioural tests carried out with the adult animals, including tests for motor activity and the swim maze, did not yield noticeable findings (Eriksson et al. 2001).

Groups of 14 to 16 male ddY mice were given a single tetrabromobisphenol A dose of 0, 0.1, 5 or 250 mg/kg body weight in corn oil. The results of behavioural tests, the open field test in addition to contextual fear conditioning and Y maze tests revealed effects at the 2 lower doses, but not at the high dose, 3 hours after dose administration (Nakajima et al. 2009).

#### 5.1.3 Dermal application

Tetrabromobisphenol A was applied to the abraded skin of groups of 5 male and 5 female New Zealand White rabbits in a dose of 2000 mg/kg body weight. The test substance was moistened with physiological saline and applied occlusively to the skin for 24 hours. Slight erythema and oedema were observed in 1 male animal after exposure. None of the animals died. No other effects were noticeable during the 14-day observation period. No effects were revealed at necropsy. Therefore, the dermal LD_50_ was above 2000 mg/kg body weight (EU 2006).

The substance was applied semi-occlusively for 24 hours to the shaved, intact or abraded skin of groups of 2 albino rabbits in doses of 1000, 2150, 4640 or 10 000 mg/kg body weight. The animals were then observed for 14 days. One animal died after 1000 and 4640 mg/kg body weight. Decreased body weights were observed at the 2 high doses. The LD_50_ was therefore above 10 000 mg/kg body weight (Hill Top Research Inc. 1966, pp. 10–20).

A tetrabromobisphenol A dose of 200 mg/kg body weight was applied to the shaved skin of 10 female rabbits for 24 hours. Erythema was observed in all animals at the end of exposure. However, this subsided by the end of the 48-hour observation period. None of the animals died and there were no other noticeable effects (no other details; EU 2006).

Tetrabromobisphenol A was applied for 24 hours to the shaved, intact skin of albino rabbits in doses of up to 3160 mg/kg body weight. No local or systemic effects were observed and neither the analysis of the urine, blood and body weights nor necropsy yielded any findings (no other details; WHO 1995).

The dermal LD_50_ was above 1000 mg/kg body weight in guinea pigs (strain not specified) (WHO 1995).

### 5.2 Subacute, subchronic and chronic toxicity

#### 5.2.1 Inhalation

Groups of 5 male and 5 female Charles River CD rats were exposed whole-body to tetrabromobisphenol A dust in concentrations of 0, 2000, 6000 or 18 000 mg/m^3^ for 4 hours a day, on 5 days a week, for 14 days. The control animals were exposed to air. Severe salivation, clear or red nasal discharge and severe lacrimation were observed at concentrations of 6000 mg/m^3^ and above. Mortality did not occur. Likewise, no substance-induced effects on body weights and feed consumption were observed and the haematological and clinico-biochemical examinations and urinalysis did not yield any findings. Gross pathological and histopathological examinations of about 21 organs did not reveal any noticeable effects. At the low concentration and above, a statistically significant decrease in the relative liver weights was observed in the female animals. The absolute liver weights were not decreased with statistical significance (International Research and Development Corporation 1975, pp. 224–243). The concentration of 18 000 mg/m^3^ is about equivalent to a dose of 3500 mg/kg body weight and day (respiratory minute volume: 0.8 l/min/kg body weight, absorption: 100%). However, the concentration in the air within the exposure chamber was not determined. Oral absorption cannot be ruled out. A NOAEC was therefore not derived.

A study investigated the hepatotoxic effects of whole-body exposure of male CD-1 mice to tetrabromobisphenol A aerosol. Groups of 20 animals were exposed to the solvent control (5% dimethyl sulfoxide) or a tetrabromobisphenol A concentration of 16 µg/m^3^ for 8 hours a day, on 6 days a week, for 60 days. The concentration was not verified by analysis. Instead, the nominal concentration obtained from a 1.6 µg/ml solution of tetrabromobisphenol A was used. Tetrabromobisphenol A led to an increase in the absolute and relative liver weights (26% and 9%, respectively), to an enlargement of the hepatocytes with significant nucleoplasm separation and increasing focal necrosis of the hepatocytes and to an increase in the activity levels of aspartate aminotransferase, ALT and alkaline phosphatase (ALP) in serum. Tetrabromobisphenol A concentrations in the liver increased from about 14 ng/g wet weight in the control group (taken from a figure) to about 35 ng/g wet weight in the animals exposed to tetrabromobisphenol A (Chen et al. 2016). Only one tetrabromobisphenol A concentration was tested. The tetrabromobisphenol A concentration of 16 µg/m^3^ is equivalent to a dose of about 12 µg/kg body weight (respiratory minute volume: 1.5 l/min/kg body weight, absorption: 100%). Conversely, in a 14-week gavage study with B6C3F1/N mice, no histological changes in the liver were observed up to a dose of 1000 mg/kg body weight and day (NTP 2014). However, as the test animals belonged to another mouse strain, the results cannot be compared directly. The dose per kg body weight calculated from the concentration is lower by a factor of 300 000 than the dose given to rats in the inhalation study described above (12 µg/kg body weight and day, 3500 mg/kg body weight and day). This calls the results of the study into question.

#### 5.2.2 Oral administration

The studies of the effects induced by tetrabromobisphenol A after repeated oral administration are shown in Table 1.

**Tab.1:** Toxicity of tetrabromobisphenol A after repeated oral administration

**Species,** **strain,** **number per group**	**Exposure**	**Findings^a)^**	**References**
**rat**, Wistar (HsdCpb:WU), 10 ♂, 10 ♀	28 days, OECD TG 407, 0, 30, 100, 300 mg/kg body weight and day, diet, purity: > 98%	**30 mg/kg body weight**: **NOAEL (♂)**; **100 mg/kg body weight and above**: ♂: plasma: total T4 ↓ (BMDL 48 mg/kg body weight); **300 mg/kg body weight**: **NOAEL (♀)**; ♂: plasma: total T3 ↑ (BMDL 124 mg/kg body weight)	van der Ven et al. 2008
**rat**, Crj:CD(SD)IGS, 0, 200, 1000 mg/kg body weight and day: 12 ♂, 12 ♀, 8, 40 mg/kg body weight and day: 6 ♂, 6 ♀	28 days, similar to OECD TG 407, 0, 8, 40, 200, 1000 mg/kg body weight and day, gavage, in corn oil, 5 days/week, purity: 99.5%	**1000 mg/kg body weight**:** NOAEL**	MHLW 2019 d, e
**rat**, Charles River CD, 25 ♂, 25 ♀	28 days, 0, 1, 10, 100, 1000 mg/kg feed (about 0, 0.12, 1.2, 12, 120 mg/kg body weight and day^b)^), diet, purity: no data	**120 mg/kg body weight**:** NOAEL**	International Research and Development Corporation 1972, pp. 244–288
**rat**, Sprague Dawley, 9 ♂	30 days, beginning on PND 18, 0, 125, 250, 500 mg/kg body weight and day, gavage, in corn oil, 7 days/week, purity: 97%	**125 mg/kg body weight**:** NOAEL**; **250 mg/kg body weight and above**: serum: total T4 ↓; **500 mg/kg body weight**: absolute and relative liver weights ↑; absolute and relative thyroid weights ↓	Choi et al. 2011
**rat**, CD Crl: CD (SD) IGS BR, 100, 300 mg/kg body weight and day: 15 ♂, 15 ♀, 0, 1000 mg/kg body weight and day: 5 ♂, 5 ♀	13 weeks, observation 6 weeks, OECD TG 408, 0, 100, 300, 1000 mg/kg body weight and day, gavage, in corn oil, 7 days/week, purity: 99%	**no NOAEL**; **100 mg/kg body weight and above**: ♂ and ♀: blood: total T4 ↓ without dose dependency (♂: days 33 and 90, ♀: only day 33, reversible), ♂: serum: ALP activity ↑ (+ 26% at end of exposure, reversible); **300 mg/kg body weight and above**: ♀: serum: total bilirubin ↑ (+ 31% at end of exposure, reversible), ALP activity ↑ (+ 26% at end of exposure, reversible), ♂: absolute spleen weights ↓ (reversible at 1000 mg/kg body weight); **1000 mg/kg body weight**: ♂: blood: thrombocyte count ↓ (20.9% at end of exposure, reversible), serum: total bilirubin ↑ (+ 78% at end of exposure, reversible)	Osimitz et al. 2016
**rat**, Sprague Dawley, 0, 100 mg/kg body weight and day: 21 ♂, 21 ♀, 0.3, 3 mg/kg body weight and day: 7 ♂, 7 ♀	13 weeks, 0, 0.3, 3, 30, 100 mg/kg body weight and day, diet, purity: no data	**100 mg/kg body weight**: ♂: blood: haematocrit ↓ (at end of exposure, but without statistical significance)	EU 2006
**rat**, F344/NTac, 10 ♂, 10 ♀	14 weeks, OECD TG 408, 0, 10, 50, 100, 500, 1000 mg/kg body weight and day, gavage, in corn oil, 5 days/week, purity: 99%	**50 mg/kg body weight**:** NOAEL**; **100 mg/kg body weight and above**: ♂ and ♀: blood: total T4 ↓ with dose dependency (♂: week 14, ♀: day 4), serum: ALT activity, SDH activity ↓ (week 14); **500 mg/kg body weight and above**: ♂ and ♀: blood: total T4 ↓ (day 4, day 23), serum: haematocrit, haemoglobin, erythrocyte count ↓ (day 23, normalization week 14), ♂: ALP activity ↑ (week 14), absolute and relative liver weights ↑, absolute and relative spleen weights ↓, ♀: ALP activity ↑ (day 4)	NTP 2014
**rat**, Wistar Han [Crl:WI(Han)], 0, 1000 mg/kg body weight and day: 60 ♂, 60 ♀, 250, 500 mg/kg body weight and day: 50 ♂, 50 ♀	♂: 104 weeks, ♀: 105 weeks, 0, 250, 500, 1000 mg/kg body weight and day, gavage, in corn oil, 5 days/week, purity: > 99%	**no NOAEL**; **250 mg/kg body weight and above**: ♀: uterus: atypical endometrial hyperplasia; **500 mg/kg body weight and above**: ♂: body weights ↓ (after 25 weeks, by at least 10%), ♀: ovaries: rete ovarii cysts (see Section 5.2.2.1) **1000 mg/kg body weight**: interim examination at an age of 3 months, no pathological findings in uterus	Dunnick et al. 2015; NTP 2014
**mouse**, Crlj:CD1 (ICR), dose groups: 8 ♂, control group: 7 ♂	2 weeks, 0, 350, 700, 1400 mg/kg body weight and day, gavage, in olive oil, 7 days/week, purity: 99.1%	**no NOAEL**; **350 mg/kg body weight and above**: ♂: liver: incidence of inflammatory cell infiltration ↑ (no dose dependency, severity not specified); **1400 mg/kg body weight**: ♂: absolute liver weights ↑ (23%), relative liver weights ↑ (17%), liver: incidence of focal necrosis of the hepatocytes ↑	Tada et al. 2007
**mouse**, B6C3F1/N, 10 ♂, 10 ♀	14 weeks, OECD TG 408, 0, 10, 50, 100, 500, 1000 mg/kg body weight and day, gavage, in corn oil, 5 days/week, purity: 99%	**100 mg/kg body weight**:** NOAEL ♂**; **500 mg/kg body weight and above**: ♂: absolute and relative liver weights ↑ (14%–19%), kidneys: incidence of cytoplasmic changes in the tubules ↑; **1000 mg/kg body weight**: ♂: absolute and relative kidney weights ↓, absolute and relative spleen weights ↑, kidneys: severity of cytoplasmic changes in the tubules ↑, ♀: absolute and relative liver weights ↑ (12%)	NTP 2014
**mouse**, B6C3F1/N, 50 ♂, 50 ♀	105 weeks, 0, 250, 500, 1000 mg/kg body weight and day, gavage, in corn oil, 5 days/week, purity: > 99%	**no NOAEL**; **250 mg/kg body weight and above**: ♂: liver: eosinophilic foci, kidneys: cytoplasmic changes in the tubules, nephropathy, ♀: forestomach: ulcer, mononuclear cell infiltration, inflammation, epithelial hyperplasia; **500 mg/kg body weight**: ♂: liver: clear cell foci, forestomach: ulcer, mononuclear cell infiltration, inflammation, epithelial hyperplasia; **1000 mg/kg body weight**: mortality ↑, ♀: body weights ↓ (after 25 weeks, by at least 10%) (see Section 5.2.2.2)	Dunnick et al. 2015; NTP 2014

ALP: alkaline phosphatase; ALT: alanine aminotransferase; BMDL: lower limit of the 95% confidence interval of the benchmark dose; PND: postnatal day; SDH: sorbitol dehydrogenase; T3: triiodothyronine; T4: thyroxine; TG: test guideline

^a)^ The described changes are statistically significant unless specified otherwise.

^b)^ conversion factor 0.12 (subchronic) according to EFSA Scientific Committee (2012)

##### 5.2.2.1 Rat

In a 28-day feeding study carried out according to OECD Test Guideline 407 in male and female Wistar (HsdCpb:WU) rats, the total T4 concentrations in the plasma of the male animals were reduced at 100 mg/kg body weight and day and above. At 300 mg/kg body weight and day, the total T3 concentrations were increased in the male animals. No other toxic effects were observed (van der Ven et al. 2008). The NOAEL for decreased total T4 concentrations was 30 mg/kg body weight and day in the males and 300 mg/kg body weight and day, the highest dose tested, in the females.

A study published in Japanese, but with English abstract and tables, reported that no toxic effects were induced up to the highest dose tested of 1000 mg/kg body weight and day after gavage administration in male and female Crj:CD(SD)IGS rats for 28 days. The study was carried out according to a test guideline of the Chemical Substances Control Law of Japan (MHLW 2019 d, e). The scope of this guideline is similar to that of OECD Test Guideline 407. A NOAEL of 1000 mg/kg body weight and day, the high dose, was derived.

Another 28-day feeding study in male and female Charles River CD rats likewise did not find toxic effects up to the highest dose tested of 1000 mg/kg body weight and day (International Research and Development Corporation 1972, pp. 244–288). However, the report included only the parameters body weights, feed consumption, behaviour, clinical symptoms and organ weights of about 10 organs. Furthermore, only certain organs such as the liver, kidneys and thyroid gland were examined histologically.

In a 28-day study with male Sprague Dawley rats, body weight gains were delayed at doses of 0.3 mg/kg body weight and day and above and haematocrit levels were reduced at 10 mg/kg body weight and day and above (Sato et al. 1996). No histopathological examinations were carried out and only 3 animals per dose were tested. Therefore, the study is not included in the evaluation.

Male Sprague Dawley rats were treated by gavage with tetrabromobisphenol A for 30 days beginning at an age of 18 days. The total T4 concentrations in serum were reduced at 250 mg/kg body weight and day and above. No substance-induced changes in survival, clinical symptoms, TSH and organ histology were observed (Choi et al. 2011). The study has a limited scope of examination because only the liver, kidneys, thyroid glands and testes were examined by histology.

In a 13-week gavage study carried out according to OECD Test Guideline 408 in CD Crl: CD (SD) IGS BR rats, changes were evident in individual parameters of the exposed animals; these changes were not dependent on the dose and were reversible after the 6-week recovery period. The total T4 concentrations were reduced in the males and the females at doses of 100 mg/kg body weight and day and above. The T3 and TSH levels remained unchanged up to the highest dose tested. The authors derived a NOAEL of 1000 mg/kg body weight and day (Osimitz et al. 2016). The decrease in the total T4 concentrations is consistent with the findings reported by several other studies (NTP 2014; van der Ven et al. 2008). As a result, the effect is attributed to the substance. A NOAEL was not derived from the study findings.

Another 13-week feeding study was carried out with Sprague Dawley rats. The original report is not available for this study. In the study, the values were not changed with statistical significance after exposure to tetrabromobisphenol A up to the high dose of 100 mg/kg body weight and day (EU 2006). The liver, kidneys, skeletal muscles and adipose tissues were examined histologically.

A study of the NTP carried out according to OECD Test Guideline 408 with 14-week gavage administration in F344/NTac rats found a dose-dependent decrease in total T4 concentrations in male and female rats at doses of 100 mg/kg body weight and day and above. The histological examination did not reveal any changes (NTP 2014). The NOAEL of this study was 50 mg/kg body weight and day because of the reduced total T4 concentrations observed at doses of 100 mg/kg body weight and day and above.

In a 2-year carcinogenicity study carried out by the NTP with gavage administration in Wistar Han rats, atypical hyperplasia in the uterine endometrium was observed in the females at the low dose of 250 mg/kg body weight and day and above (see Section 5.7.2; Dunnick et al. 2015; NTP 2014). It is therefore not possible to derive a NOAEL.

A study in juvenile rats given oral doses from postnatal days 4 to 21 (Fukuda et al. 2004) is not used for evaluation because its findings are not relevant to the workplace.

##### 5.2.2.2 Mouse

In a 2-week gavage study in male Crlj:CD1 (ICR) mice, the incidence of inflammatory cell infiltration in the liver was increased without dose dependency at the lowest dose tested of 350 mg/kg body weight and day and above. Mortality was not observed. Body weights, clinical symptoms, haematology and serum biochemistry were unchanged up to the highest dose of 1400 mg/kg body weight and day (Tada et al. 2007). As the effects were not dependent on the dose and were not observed in studies with longer periods of exposure, these effects are not considered relevant to the evaluation.

A 14-week gavage study carried out by the NTP in male and female B6C3F1/N mice according to OECD Test Guideline 408 found an increase in liver weights and in the incidence of cytoplasmic changes in the renal tubules of male mice at doses of 500 mg/kg body weight and day and above (NTP 2014). A NOAEL of 100 mg/kg body weight and day was derived from this study.

A 2-year carcinogenicity study with gavage administration in B6C3F1/N mice (see Section 5.7.2) found an increased incidence of eosinophilic foci in the liver and cytoplasmic changes in the renal tubules of the male animals and an increased incidence of ulcers, mononuclear cell infiltration, inflammation and epithelial hyperplasia in the forestomach of the female animals at the lowest dose tested of 250 mg/kg body weight and day and above (Dunnick et al. 2015; NTP 2014). As a result, it was not possible to derive a NOAEL.

###### 5.2.2.2.1 Neurotoxicity

In a 2-week gavage study, 4 to 8 male C57BL/6 mice (6 weeks old) per dose group were given daily doses of tetrabromobisphenol A (purity: 97%) of 0, 20, 100 or 500 mg/kg body weight and day in corn oil. At doses of 100 mg/kg body weight and day and above, survival of new cells in the dentate gyrus of the hippocampus was decreased in a dose-dependent manner (cell proliferation was not impaired) and the latency period in the passive avoidance test, an assay that provides a means of assessing learning memory, was likewise decreased in a dose-dependent manner. On day 2, the latency period was reduced at doses of 100 and 500 mg/kg body weight and day; on day 8, however, the effects were observed only at a dose of 500 mg/kg body weight and day. The microglia and astrocytes were activated in a dose-dependent manner at 100 mg/kg body weight and day and above; this was regarded as the expression of an inflammatory change. The concentration of brain-derived neurotrophic factor (BDNF) was reduced in hippocampus homogenate at 500 mg/kg body weight and day. Immunohistochemical analysis revealed that the CREB protein was reduced in the hippocampus at 500 mg/kg body weight and day. A histological examination of the hippocampus (form, neuronal density, neuronal damage/loss) as well as the Morris water maze test did not reveal any noticeable effects up to the high dose (Kim et al. 2017). A NOAEL of 20 mg/kg body weight and day was determined for the reduced survival of new cells in the dentate gyrus of the hippocampus. The study has methodological ambiguities, such as discrepancies in the number of examined animals. No data for systemic toxicity were provided, including body weights, feed consumption and clinical findings. According to the Commission, it is a neurobiological study that fulfils most of the conventional standards. Neuroscientific studies that investigate neurogenesis (the generation of new neurons) in the hippocampus use mainly male animals because of possible covariables such as varying oestrogen levels in groups made up only of female animals. The proliferation data must be interpreted with caution because a BrdU pulse-chase experiment with the sacrifice of the animals 24 hours after administration of the last BrdU dose is not available. Instead, the analysis was carried out after 9 days at a stage when the neuroblasts were immature. In addition, specific markers for neurogenesis were not analysed, allowing also other cells such as astrocytes to proliferate. Other animals were used for the BDNF-ELISA. However, findings were reported only for the high dose of 500 mg/kg body weight and day. The results of the passive avoidance test revealed a dose-dependent retention deficit on day 2, which persisted on day 8 at a dose of 500 mg/kg body weight and day. However, the number of examined animals was very small. At least 16 animals are required for the test to be considered valid. The Morris water maze test, on the other hand, yielded negative results. A valid 2-generation study carried out in rats according to OECD Test Guideline 416 obtained negative results in the F2 offspring in several neurophysiological tests and behavioural tests at doses up to 1000 mg/kg body weight and day (Cope et al. 2015). Therefore, the findings missing from the behavioural tests are not consistent with the changed functional markers. As a result of this and the uncertainties in the neurogenetic assessment, the study has limited validity. For this reason, the study is not suitable for the derivation of a MAK value.

After tetrabromobisphenol A was given to 10 ICR mice in an oral dose of 250 mg/kg body weight for 30 days, the auditory brainstem response was increased in comparison with the values determined in the control animals. This was regarded as evidence of hearing loss (Park et al. 2016). No data were available for the sex of the animals, the route of administration (probably gavage), the frequency of administration, the vehicle and the purity of the substance. In vitro tests that were carried out simultaneously reported an increased incidence of apoptosis in the HEI-OC1 auditory cell line and in Corti’s organ explants from Sprague Dawley rats. This suggests that there may be effects on hearing.

##### 5.2.2.3 Summary

Based on the findings of a 14-week study with gavage administration, a NOAEL of 50 mg/kg body weight and day was derived for decreased thyroxine concentration, the most sensitive end point, in male and female F344/NTac rats. The LOAEL (lowest observed adverse effect level) was 100 mg/kg body weight and day (NTP 2014). The 14-week study with gavage administration in male B6C3F1/N mice established the cytoplasmic changes in the renal tubules that occurred at 500 mg/kg body weight and day and above as the critical end point. The NOAEL for this effect was 100 mg/kg body weight and day (NTP 2014).

It was not possible to derive NOAELs for Wistar Han rats and for B6C3F1/N mice from the findings of long-term carcinogenicity studies (see also Section 5.7.2). Atypical endometrial hyperplasia was detected in Wistar Han rats at doses of 250 mg/kg body weight and day and above (Dunnick et al. 2015; NTP 2014). Histological examinations of the uterus carried out in studies with shorter periods of exposure of up to 14 weeks did not reveal any noticeable findings in CD [Crl: CD (Sprague Dawley) IGS BR], F344/NTac and CD Crl: CD (SD) IGS BR rats after exposure to doses of up to 1000 mg/kg body weight and day. The same was reported by a carcinogenicity study in Wistar Han rats at interim necropsy after 3 months (Cope et al. 2015; NTP 2014; Osimitz et al. 2016). A long-term study found eosinophilic foci in the liver of B6C3F1/N mice at doses of 250 mg/kg body weight and day and above (Dunnick et al. 2015; NTP 2014). The lowest NOAEL for liver effects to be derived from the findings of short-term studies is the NOAEL of 100 mg/kg body weight and day that was obtained from a 14-week study for an increase in liver weights in male mice of less than 20% (an increase in liver weights of less than 20% is not regarded as adverse) (LOAEL: 500 mg/kg body weight and day; NTP 2014). The histological examinations of the liver carried out in the short-term studies and at interim necropsy in the long-term study did not reveal noticeable effects up to a dose of 1000 mg/kg body weight and day, the highest dose tested (NTP 2014).

#### 5.2.3 Dermal application

Tetrabromobisphenol A was applied non-occlusively as a paste to the shaved dorsal skin of groups of 4 male and 4 female New Zealand White rabbits in doses of 0, 100, 500 or 2500 mg/kg body weight and day (no data for purity) in 0.9% physiological saline on 5 days a week for 3 weeks. The animals wore a collar. Mortality and toxic effects were not observed. Very mild erythema developed in some cases at 100 mg/kg body weight and day. Almost all animals had mild erythema at 500 and 1500 mg/kg body weight and day. No unusual findings were detected for body weights, haematological and clinico-chemical parameters and urinalysis. Necropsy and the histopathological examination of about 30 organs did not reveal any effects. No substance-related changes in organ weights were observed (International Research and Development Corporation 1979, pp. 166–202).

### 5.3 Local effects on skin and mucous membranes

#### 5.3.1 Skin

In a patch test, 0.5 g of tetrabromobisphenol A in 0.5 ml of corn oil was applied occlusively to the intact and abraded skin of 6 albino rats for 24 hours. None of the animals exhibited signs of irritation after 24 and 72 hours. The primary irritation index was 0 (Hill Top Research Inc. 1966, pp. 10–20).

Three male and 3 female New Zealand White rabbits were administered 0.5 g of tetrabromobisphenol A. The test substance was slightly moistened with physiological saline and applied occlusively for 24 hours to 2 abraded and 2 intact areas of skin on each animal. There was no evidence of irritation 24 and 72 hours after application (EU 2006).

In another skin irritation study, 0.5 g of tetrabromobisphenol A (not specified whether moistened) was applied occlusively to the shaved dorsolateral skin of 6 albino rabbits (no other details) for 24 hours. The skin of 3 animals was abraded and that of the other 3 was not abraded. The application sites were examined 24 and 72 hours after application and scored using the Draize scale. No signs of irritation were observed at the intact skin sites. For the animals with abraded skin, the mean scores for oedema were 1 and 0 at 24 and 72 hours, respectively, and the mean scores for erythema were 0 and 0.3 at 24 and 72 hours, respectively (EU 2006).

In a range-finding study, tetrabromobisphenol A was applied undiluted to the intact and abraded skin of a single rabbit (no other details) and as a 10% solution in dipropylene glycol monomethyl ether to the intact and abraded skin and to the skin on the ear of a second rabbit. The test substance was applied to the abraded skin on 3 consecutive days and to the intact skin on 10 consecutive days. Neither the diluted nor the undiluted substance had an effect on the intact skin. Scabbing and scarring were observed on the abraded skin after application of both the undiluted substance and the 10% solution. It remained unclear whether these were a consequence of the abrasion procedure (EU 2006).

A 4-week study, in which 0.1 ml of a 0.5%, 5% or 50% tetrabromobisphenol A solution was applied to the skin on the ears of New Zealand White rabbits, did not report signs of “bromide acne” (no other details; EU 2006).

**Conclusion**: Tetrabromobisphenol A does not induce irritation of the skin in rabbits and rats.

#### 5.3.2 Eyes

In a study, 100 mg of tetrabromobisphenol A was instilled into the conjunctival sac of the right eye of 3 male and 3 female New Zealand White rabbits. The eyes were examined 1, 24, 48 and 72 hours and 7 days after application. Slight conjunctival redness (grade 1) was observed in 4 animals after 1 hour; this effect was no longer noticeable after 24 hours. No other signs of irritation were observed at any other time point (EU 2006).

In another study, 100 mg of tetrabromobisphenol A was instilled into the conjunctival sac of the right eye (no other details) of 6 male albino rabbits (no other details). Soon after the instillation of the test substance, lacrimation and conjunctival erythema were observed; these effects had completely disappeared after 24 hours. The eyes were examined 24, 48 and 72 hours and 7 days after instillation. After 48 hours, mean scores of 0.17 for conjunctival redness and iritis and a mean score of 0.3 for conjunctival chemosis were determined. After 72 hours, scores of 0.17 were obtained for conjunctival chemosis and iritis. All animals had scores of 0 on day 7 after application (EU 2006).

Tetrabromobisphenol A was instilled in an amount of 100 mg into the left eye of 6 albino rabbits. The right eye served as the control. The eyes were examined after 24, 48 and 72 hours. Mean scores of 1.5, 0.5 and 0.5, respectively, were determined for conjunctival erythema using the Draize method. The results were assessed as borderline and the test was repeated with 6 other animals. The mean irritation scores were 0.8, 0.17 and 0 (Hill Top Research Inc. 1966, pp. 10–20).

Tetrabromobisphenol A was instilled in an amount of 3 mg into 1 eye of 3 New Zealand White rabbits. The eyes were examined 5 minutes, 1 hour and 4 hours after instillation and then on a daily basis up to 7 days after instillation. Irritation was not observed in the cornea, iris or conjunctiva. An irritation score of 0 was determined using the Draize method (no other details; International Bio-Research Inc. 1967 a, pp. 208–210).

Tetrabromobisphenol A was instilled into both eyes of 2 rabbits (no other details, 1 rabbit per test condition) either in undiluted form (probably as a powder) or as a 10% suspension. The right eye of each animal was washed (time point not specified), the left eye remained unwashed. In both the washed and unwashed eyes, the undiluted test substance caused immediate, but very slight conjunctivitis (grade 2) which diminished to grade 1 within 1 hour and disappeared within 48 hours. In both the washed and the unwashed eyes, the 10% solution caused “slight pain”, conjunctivitis and “corneal damage” (no other details) for 3 days. The eyes returned to normal within a week (EU 2006).

**Conclusion**: Tetrabromobisphenol A does not cause irritation of the eyes in rabbits.

### 5.4 Allergenic effects

#### 5.4.1 Sensitizing effects on the skin

The REACH dossier includes a modified Buehler test. In this test, induction was performed by occlusive application of 500 mg of tetrabromobisphenol A (moistened with 80% ethanol) for a total of 9 treatments instead of the 3 recommended by OECD Test Guideline 406. Furthermore, only 10 female Hartley guinea pigs were tested instead of the recommended 20. The same test formulation was used both for the induction treatment and for the 2 challenge treatments that were carried out at an interval of 48 hours. A reaction was not noticeable in any of the animals (ECHA 2019; EU 2006).

A Landsteiner-Draize test was carried out with 10 intradermal injections of a 0.1% test formulation of tetrabromobisphenol A in 0.9% sodium chloride solution. The challenge treatment was performed using the same procedure and took place 2 weeks after the last induction treatment. A reaction was not observed in the 8 guinea pigs tested (International Research and Development Corporation 1978 b, pp. 122–132).

#### 5.4.2 Sensitizing effects on the airways

There are no data available.

### 5.5 Reproductive and developmental toxicity

#### 5.5.1 Fertility

The generation studies that were carried out with tetrabromobisphenol A are shown in Table 2.

**Tab.2:** Generation studies with tetrabromobisphenol A

**Species, strain, number per group**	**Exposure and examination time points**	**Findings**	**References**
**rat**, CD [Crl: CD (Sprague Dawley) IGS BR], 30 ♂, 30 ♀	**2-generation study**, OECD TG 416, deviation from TG: F2 animals not sacrificed after PND 21, exposure: 10 weeks before mating, during mating and gestation up to PND 21, 0, 10, 100, 1000 mg/kg body weight and day, gavage, 7 days/week, purity: no data, probably 98.91% (same as MPI Research (2001)), vehicle: corn oil, litters reduced to 8 (F1) or 10 offspring (F2) with the same number per sex, examination: parental generation: ♂ after mating, ♀: PND 21, F1 generation: same as F0 generation, F2: brain weights, neuropathology and FOB (PND 11, 60)	**10 mg/kg body weight**:** NOAEL parental toxicity**; **at 100 mg/kg body weight and above**: F0 ♂: serum: total T4 ↓, F1: serum: total T4, total T3 ↓; **1000 mg/kg body weight**:** NOAEL fertility, perinatal toxicity**; F0 ♂: serum: total T3 and total T4 ↓, F0 ♀: serum: total T4 ↓, F1: total sperm concentration/cauda epididymis ↓ (sperm motility and percentage of abnormal sperm not negatively affected, therefore of questionable relevance), F2: brain: reduced thickness of the parietal cortex (PND 11; no histological findings, no adverse effects in behavioural tests, therefore, unknown biological relevance), no noticeable changes in: oestrus cycle (F0, F1), histology of the uterus (F0, F1; both uterine horns examined), ♂/♀: onset of puberty and AGD (F1, F2), no oestrogenic effects in gross anatomy and microanatomy, fertility, reproduction	Cope et al. 2015
**rat**, Wistar (HsdCpb:WU), 10 ♂, 10 ♀	**1-generation study**, based on OECD TG 415, F0 generation: beginning 70 (♂) or 14 (♀) days before mating, during mating (♂) and gestation, up to weaning on PND 21 (♀), F1 generation: up to weaning on PND 21, 0, 3, 10, 30, 100, 300, 1000, 3000 mg/kg body weight and day, feed, purity: > 98%, examination: F0 generation: ♂: after mating, ♀: PND 21, F1 generation: PND 21 (2 ♂, 2 ♀) and at 10 weeks of age	**evaluation based on calculated values**:** CED;** **1.4 mg/kg body weight**: CED: F1 ♂: testis weights ↑ (BMDL: 0.5 mg/kg body weight); **2.2 mg/kg body weight**: CED: F1 ♂: pituitary gland weights ↑ (BMDL: 0.6 mg/kg body weight); **36 mg/kg body weight**: CED: F1 ♀: total T4 in plasma ↓ (BMDL: 16 mg/kg body weight); **100 mg/kg body weight**: CED: F1 ♂: total T4 in plasma ↓ (BMDL: 31 mg/kg body weight); **2993 mg/kg body weight**: CED: F1 ♀: delayed onset of puberty (vaginal opening; BMDL 2745 mg/kg body weight); **3000 mg/kg body weight**:** NOAEL fertility and perinatal toxicity**; no statistical evaluation with pairwise comparisons with the controls, F1: 1^st^ cluster of correlating parameters: reduced total T4 with developmental parameters (♀: delayed sexual development, offspring mortality ↓, effects on brainstem auditory evoked potentials (Lilienthal et al. 2008)), 2^nd^ cluster of correlating parameters: gonad weights ♂ with gonad weights ♀, ♀ endometrial thickness, ♀ CYP19 in ovaries, ♂ testosterone in plasma; no noticeable changes in: plasma: testosterone and oestradiol ♂ (♀ not examined), ♀, ♂ onset of puberty, ♀ oestrus cycle	van der Ven et al. 2008
see above	see above examination on PND 50 to 110: brainstem auditory evoked potentials, tests for context or cue conditioned fear or sweet preference	brainstem auditory evoked potentials: **6.6 mg/kg body weight**: CED: F1 ♀: threshold ↑ (2 kHz; BMDL: 0.9 mg/kg body weight); **36.3 mg/kg body weight**: CED: F1 ♂: prolongation of wave IV latency (0.5 kHz; BMDL: 7.7 mg/kg body weight); **61.0 mg/kg body weight**: CED: F1 ♂: prolongation of interpeak latencies II–IV (2 kHz; BMDL: 22.9 mg/kg body weight); **70.3 mg/kg body weight**: CED: F1 ♀: prolongation of wave IV latency (2 kHz; BMDL: 8.3 mg/kg body weight)	Lilienthal et al. 2008

AGD: anogenital distance; BMDL: lower limit of the 95% confidence interval of the benchmark dose (CED); CED: critical effect dose (= benchmark dose at a 5% to 10% change); FOB: functional observational battery; PND: postnatal day; T3: triiodothyronine; T4: thyroxine; TG: test guideline

In a 2-generation study carried out with CD [Crl: CD (Sprague Dawley) IGS BR] rats according to OECD Test Guideline 416, no effects on fertility occurred up to 1000 mg/kg body weight and day. The NOAEL for fertility and perinatal toxicity was 1000 mg/kg body weight and day, the highest dose. In the parent generations, the T4 concentrations were decreased in the male animals at doses of 100 mg/kg body weight and day and above. The NOAEL for this effect was 10 mg/kg body weight and day. The authors do not consider the decreased T4 concentrations as relevant to humans and attribute this effect to the induction of T4-degrading enzymes such as UGT (not investigated) (Cope et al. 2015). This explanation is not plausible because gavage treatment of F344/NTac rats for 14 weeks with doses of up to 1000 mg/kg body weight and day did not lead to the induction of UGT even though T4 concentrations were reduced (NTP 2014).

A 1-generation study in Wistar (HsdCpb:WU) rats was evaluated using the PROAST software for dose–response modelling. The critical effect dose (CED) was calculated from the best fitting curve at a significance level of < 5% (equivalent to the benchmark dose). The correlation coefficients were based on group averages instead of individual comparisons. This method enables comparisons to be made between the age cohorts and sexes and ignores variability within groups. For this reason, the correlations should be regarded as indicators for clustering. A statistical test using pairwise comparisons was not included. End points for fertility such as perinatal toxicity (mortality and body weights up to postnatal day 4) did not reveal substance-induced changes (see Section 5.5.2; Lilienthal et al. 2008; van der Ven et al. 2008). The NOAEL for fertility and perinatal toxicity was 3000 mg/kg body weight and day. A critical analysis of this study found methodological confounding factors. The thresholds of the brainstem auditory evoked potentials and the latency had no consistent pattern and are inconsistent with the anatomy and physiology of the auditory system. Possible explanations for the differences in the thresholds and the latency are hypothermia or the use of immature test animals. In addition, the groups were made up of only 5 to 6 animals. The CEDs were not reproducible when modelled using the BMDS software of the US EPA. This argues against the effects having any biological significance (Strain et al. 2009).

In a 2-generation study in CD1 mice, the substance was administered with the drinking water in the only dose tested of 35 µg/kg body weight and day. Morphometric effects were observed in the seminiferous tubules of the testes in the F1 offspring of exposed dams. These effects were likewise observed in a second generation born of treated dams mated with untreated or treated male mice, but not in those born of untreated dams mated with treated male mice. The germinal epithelial thickness was decreased and the number of apoptotic cells was increased. Sperm parameters and reproduction were not impaired (Zatecka et al. 2013). Only a very low dose was tested. As a result, it is not possible to derive a dose–response relationship and it is difficult to interpret the changes of the parameters. In addition, a 2-generation study with rats did not reveal histological effects on the male reproductive organs at a dose of 1000 mg/kg body weight and day, a dose that was almost 30 000 times as high (Cope et al. 2015). As a result, the effects in mice seem questionable. The study is therefore not included in the evaluation.

The uterus was the focus of a gavage study with 14 or 28-day administration of tetrabromobisphenol A in doses of 0, 5 or 50 mg/kg body weight and day to groups of 8 female BALB/c mice per exposure period and dose. Oedema, inflammatory infiltrates, an increase in the number of vessels, more severe damage to the endometrium and an increased endometrial thickness were observed in the uterus at doses of 5 mg/kg body weight and day and above. Increased levels of interleukin-2 (IL-2) (14 and 28 days) and tumour necrosis factor α (TNF-α) (28 days) were found in the serum at 50 mg/kg body weight and day (Zhang et al. 2021). However, the figures in the publication do not depict the uterine endometrium as described, but epithelial tissue, probably taken from the vaginal or cervical epithelium. The phases of the oestrus cycle were not taken into consideration, even though this information is important for the evaluation of substance effects. Analysis of the cytokines and lymphocytes yielded, in spite of a high standard deviation, statistical significances and inconsistent findings with respect to exposure duration and dose. This calls the validity of the results into question. As a result, the study is not included in the evaluation and is not suitable for the derivation of a MAK value.

#### 5.5.2 Developmental toxicity

Table 3 gives an overview of the developmental toxicity studies that investigated tetrabromobisphenol A.

**Tab.3:** Developmental toxicity studies after administration of tetrabromobisphenol A

**Species,** **strain,** **number per group**	**Exposure and examination**	**Findings**	**References**
**rat**, Charles River CD (no other data), 5 ♀	**GD 6–15**, **pilot study**, 0, 30, 100, 300, 1000, 3000, 10 000 mg/kg body weight and day, gavage, purity: no data, vehicle: corn oil, examination GD 20	**10 000 mg/kg body weight**: dams: mortality (3/5), soft, green faeces, matted hair in the anogenital area, body weight gains ↓; no unusual findings: number of living foetuses, resorptions, implantations, corpora lutea, foetuses not examined for teratogenicity	International Research and Development Corporation 1978 d, pp. 53–62
**rat**, CD [Crl: CD (Sprague Dawley) IGS BR], 25 ♀	**GD 0–19**, **OECD TG 414**, 0, 100, 300, 1000 mg/kg body weight and day, gavage, purity: 98.91%, vehicle: corn oil, examination GD 20	**1000 mg/kg body weight**:** NOAEL maternal and developmental toxicity**	Cope et al. 2015; MPI Research 2001
**rat**, Wistar, 24–26 ♀	**GD 0–19**, 0, 280, 830, 2500 mg/kg body weight and day, oral, probably gavage, purity: no data, vehicle: olive oil, examination GD 20 (14 dams and their foetuses) and PND 21 (remaining animals)	**2500 mg/kg body weight**:** NOAEL maternal and developmental toxicity**; no unusual findings: length of gestation, number of corpora lutea, implantations, surviving foetuses, late foetal deaths, foetuses: external, skeletal and visceral changes, body weights offspring: body weight gains, developmental milestones, general symptoms	EU 2006; Noda 1985
**rat**, Crj:CD(SD)IGS, 8 ♀	**GD 10–PND 20**, 0, 100, 1000, 10 000 mg/kg feed (GD 10–20: 0, 10, 87, 819 mg/kg body weight and day), purity: > 98%, PND 2: litters reduced to 4 offspring per sex and litter, examinations PND 1, PND 20, PND 77	**87 mg/kg body weight**: offspring: brain: hilus of the dentate gyrus: reelin-expressing interneurons ↑ (PND 20; no longer found on PND 77, sign of impaired neuronal migration), hilus: number of mature neurons ↑ (PND 77); **819 mg/kg body weight and above**: dams: relative uterus weights ↓ (PND 77), offspring: brain: subgranular zone: apoptotic bodies ↑ (PND 20; no longer found on PND 77, sign of impaired neurogenesis); no unusual findings: dams: body weight gains GD 10–20, number of implantation sites, number of living offspring, length of gestation, thyroid glands: weight and histology (PND 20), offspring: body weights, relative AGD, sex ratio (PND 1), organ weights (PND 20), T3, T4, TSH concentrations (10 offspring/group examined, PND 20, PND 77; T3 reduced on PND 20 at 10 and 87 mg/kg body weight, but not at 819 mg/kg body weight), onset of puberty; study not carried out according to valid test guidelines, very few animals examined	Saegusa et al. 2009, 2012
**mouse**, Crlj:CD1 (ICR), 6 ♀	**GD 0–PND 27**, 0, 100, 1000, 10 000 mg/kg feed (GD 0–17: 0, 16, 141, 1640 mg/kg body weight and day), purity: 99.1%, PND 4: litters reduced to 4 offspring per sex and litter, examination PND 27	**16 mg/kg body weight and above**: dams, offspring: kidneys: dilation of renal tubules, cysts; liver: focal necrosis of hepatocytes, inflammatory cell infiltration; **141 mg/kg body weight and above**: offspring: serum: ♂: total cholesterol ↑, ♀: triglycerides ↓; **1640 mg/kg body weight**: dams: after birth relative liver weights ↑, serum: total cholesterol ↑, triglycerides ↑; offspring: relative liver weights ↑, ♀: relative brain weights ↑, serum: ♀: total cholesterol ↑, ♂: triglycerides ↓; study not carried out according to valid test guidelines, very few animals examined	Tada et al. 2006
**mouse**, CD-1, 5 ♀	**GD 8–PND 21**, 0, 0.2 mg/kg body weight and day, with pipette, purity: 97%, vehicle: corn oil, examination: several time points using different behavioural tests	**0.2 mg/kg body weight**: effects in tests of sociability; non-standardized tests	Kim et al. 2015
**rat**, MOL:WIST, 20 ♀	**GD 7–PND 17**, basis: the OECD TG 426 proposed at that time, 0, 50, 250 mg/kg body weight and day, gavage, purity: no data, vehicle: arachis oil, examinations: histology of a small number of organs (PND 15, 22, adult), motor activity and habituation capability (PND 21, 27, 12 weeks), play behaviour (PND 31), sweet preference (5 months), Morris water maze (9, 13, 17 weeks), 8-arm radial maze (6–7 months)	**250 mg/kg body weight**: ♂: slight changes in learning and memory (Morris water maze and 8-arm radial maze, in both tests no consistent pattern if different time points compared, ♀ no changes), ♀: slight changes in habituation behaviour in test for motor activity (no consistent pattern if different time points compared, ♂ no changes); no noticeable changes: offspring: AGD (PND 0), areola and nipples (PND 13, 14), onset of puberty (♂ and ♀)	EU 2006
**rat**, Wistar Han, number of animals not specified	**GD 9–PND 21**, range-finding study, 0, 0.1 mg/kg body weight and day, feed pellets, purity: 97%, vehicle: ethanol, examinations: light–dark box (PND 110–120), elevated plus maze (PND 185–192), running wheel (PND 160–170)	**0.1 mg/kg body weight**: offspring: no noticeable changes	Rock et al. 2019
**rat**, Wistar Han, 20–24 ♀	**GD 6–PND 21 (dams)**, **PND 22–PND 90 (offspring)**, 0, 0.1, 25, 250 mg/kg body weight and day, gavage, purity: 97%, vehicle: sesame oil, examinations: open field (PND 90 and 145–150), light–dark box (PND 150–155), elevated plus maze (PND 155–160); 9–24 offspring examined per sex and time point, ♀ tested during oestrus	**25 mg/kg body weight and above**: offspring: ♂: elevated plus maze: open arm entries ↓ (measure for exploration and anxiety-like behaviour, ♀ no effects); **250 mg/kg body weight**: offspring: ♂: elevated plus maze: fraction of animals with freezing behaviour ↑ (measure for anxiety-like behaviour, ♀ no effects), positive trend: offspring: open field; ♂: light–dark box: light box entries ↓, latency to enter light box ↑ (♀ no effects)	Rock et al. 2019

AGD: anogenital distance; GD: gestation day; PND: postnatal day; T3: triiodothyronine; T4: thyroxine; TSH: thyroid-stimulating hormone

##### 5.5.2.1 Prenatal development

In a pilot study in which rats were given gavage doses of tetrabromobisphenol A from gestation days 6 to 15, 3 of 5 dams died at the high dose of 10 000 mg/kg body weight and day. The number of living foetuses, resorptions, implantations and corpora lutea remained unchanged up to the high dose. The foetuses were not examined for teratogenicity (International Research and Development Corporation 1978 d, pp. 53–62).

A prenatal developmental toxicity study was carried out with CD [Crl: CD (Sprague Dawley) IGS BR] rats according to OECD Test Guideline 414. The substance was administered by gavage from gestation days 0 to 19. No maternal toxicity or toxic effects on development were found up to the highest dose tested of 1000 mg/kg body weight and day. Teratogenicity was not observed. A NOAEL of 1000 mg/kg body weight and day, the highest dose, was derived for developmental and maternal toxicity (Cope et al. 2015; MPI Research 2001).

The original report of another study that investigated prenatal toxicity was published in Japanese, but with tables and figures in English. Wistar rats were exposed by an oral route of administration, probably gavage, from gestation days 0 to 19. On gestation day 20, 14 dams were examined together with their foetuses. The remaining dams and their offspring were examined on postnatal day 21. No changes were noticeable in the dams and no effects were found in the foetuses or offspring up to the highest dose tested of 2500 mg/kg body weight and day. A NOAEL of 2500 mg/kg body weight and day was derived for maternal and developmental toxicity (EU 2006; Noda 1985).

##### 5.5.2.2 Postnatal development

A NOAEL of 1000 mg/kg body weight and day, the highest dose, was established for perinatal toxicity based on the findings of the 2-generation study with CD [Crl: CD (Sprague Dawley) IGS BR] rats described above. On postnatal day 11, the thickness of the parietal cortex in the brain was reduced in the animals of the F2 generation that were administered a dose of 1000 mg/kg body weight and day. However, the authors advised that these results should be interpreted with caution because of the limitations of the morphometric technique used. Morphometric measurements were taken of only one section of the parietal cortex per animal, at only one time point during development and by single line transect sampling. Furthermore, several neurophysiological tests carried out with the animals of the F2 generation (forelimb and hind limb grip strength, motor activity, acoustic startle reflex, passive avoidance test, Morris water maze) at different time points did not reveal any effects. T4 concentrations were decreased in the serum of the males of the parent generation at doses of 100 mg/kg body weight and day and above. As a result, the NOAEL for parental toxicity was 10 mg/kg body weight and day (see Table 2, Section 5.5.1; Cope et al. 2015).

In the 1-generation study in Wistar (HsdCpb:WU) rats described above, no changes in perinatal parameters were observed up to the highest dose tested (see Table 2, Section 5.5.1; van der Ven et al. 2008). On this basis, a NOAEL of 3000 mg/kg body weight and day, the highest dose, was derived for perinatal toxicity.

A separate publication that evaluated the findings of this generation study detected changes in the brainstem auditory evoked potentials of the animals of the F1 generation. Effects were observed on the threshold of the brainstem auditory evoked potentials and the interpeak and wave IV latencies. The lowest critical effect dose was 6.6 mg/kg body weight and day in the females and 36.3 mg/kg body weight and day in the males. BMDLs of 0.9 mg/kg body weight and day and 7.7 mg/kg body weight and day, respectively, were calculated based on these values. The authors found that the females had mainly cochlear effects, while neuronal effects predominated in the males. According to the authors, it is well documented that an adequate replenishment of thyroid hormones is important for the development of auditory structures and their function (see Table 2, Section 5.5.1; Lilienthal et al. 2008). However, this study has not been included in the evaluation because of the shortcomings that were already described in detail in Section 5.5.1.

In a study with prenatal and postnatal administration of tetrabromobisphenol A to Crj:CD(SD)IGS rats with the feed from gestation day 10 to postnatal day 20, no findings were noticeable in the dams during gestation and in the offspring up to the highest dose tested of 819 mg/kg body weight and day. The only effect was a decrease in the relative uterine weights of the dams on postnatal day 77 (Saegusa et al. 2009); this is not regarded as a typical form of maternal toxicity. The study was not carried out according to valid test guidelines and only a very small number of animals was used. For this reason, the study has not been included in the evaluation. An immunohistochemical examination of the brain of the offspring revealed an increase in the number of reelin-expressing interneurons in the hilus of the dentate gyrus on postnatal day 20 at doses of 87 mg/kg body weight and day and above. This effect is a sign of disrupted neuronal migration, but was reversible. On postnatal day 77, the number of mature neurons in the hilus was increased at these doses. The number of apoptotic bodies in the subgranular zone was increased on postnatal day 20 in the group fed a dose of 819 mg/kg body weight and day. However, this effect was no longer observed on postnatal day 77 (Saegusa et al. 2012).

In another study with prenatal and postnatal administration of tetrabromobisphenol A to Crlj:CD1 (ICR) mice with the feed from gestation day 0 to postnatal day 27, histological changes were induced in the liver and kidneys of the dams and their offspring at doses of 16 mg/kg body weight and day and above (Tada et al. 2006). With a group size of 6 animals, the number of animals is much too small to ensure reliability. As a result, this study has not been included in the evaluation.

CD-1 mice were studied using a non-standardized test system that did not comply with valid test guidelines. Effects on sociability were found at the only tetrabromobisphenol A dose tested of 0.2 mg/kg body weight and day. However, no effects on body weights were observed (Kim et al. 2015). In general, effects on sociability are related to body weight. As only one dose was tested and the test system was not a standardized system, the study has not been included in the evaluation.

In an unpublished study (original report not available) with MOL:WIST rats that were given tetrabromobisphenol A by gavage from gestation day 7 to postnatal day 17, slight effects on learning and memory (Morris water maze and 8-arm radial maze) were observed in the male offspring and effects on habituation behaviour (motor activity tests) in the female offspring at the highest dose tested of 250 mg/kg body weight and day. In both cases, no noticeable effects were detected in the other sex. The risk assessment report concluded that there is only limited evidence of developmental neurotoxicity because the changes in the parameters were only slight, a consistent pattern cannot be found between the different time points, the changes in both sexes were not consistent and there are no relevant histopathological correlates (EU 2006).

In a study with prenatal and postnatal exposure of Wistar Han rats, male offspring exhibited reduced exploratory behaviour in the elevated plus maze test and anxiety-like behaviour at doses of 25 mg/kg body weight and day and above. These effects were not observed at 0.1 mg/kg body weight and day. The females were tested during oestrus to avoid the effects of the oestrus cycle, but did not exhibit any changes in behaviour. The open field test and the light–dark test did not yield consistent findings (Rock et al. 2019).

A review evaluated whether a relationship can be established between perinatal exposure to polybrominated diphenyl ether congeners, hexabromocyclododecane or tetrabromobisphenol A and motor activity based on animal studies. The review was based on a total of 29 studies, 2 of which investigated tetrabromobisphenol A (Eriksson et al. 2001; see Section 5.1.2; MPI Research 2001). The studies lacked consistency in terms of the type of motor activity affected (locomotion, rearing or total activity), the direction (increase or decrease), the pattern of change, the existence of a dose–response relationship, the permanency of the findings and sex differences. For this reason, no causal relationship can be established between perinatal exposure to the above-mentioned substances and changes in motor activity (Williams and DeSesso 2010).

### 5.6 Genotoxicity

#### 5.6.1 In vitro

In a large number of bacterial gene mutation assays carried out in the Salmonella typhimurium strains TA92, TA98, TA100, TA1535, TA1537 and TA1538 as well as in Escherichia coli, tetrabromobisphenol A was not mutagenic in the concentration range of 0.1 to 10 000 μg/plate either in the presence or in the absence of a metabolic activation system (EU 2006; Litton Bionetics Inc. 1976, pp. 214–223, 1977, pp. 21–32; MHLW 2019 b, e; Mortelmans et al. 1986; NTP 2014; SRI 1976 a, b).

Studies investigating other end points of in vitro genotoxicity tests are shown in Table 4.

**Tab.4:** In vitro studies of the genotoxicity induced by tetrabromobisphenol A

**End point**	**Test system**	**Concentrations**	**Cytotoxicity/Comments**	**Results^a)^**		**References**
				**–m. a.**	**+m. a.**	
non-covalent DNA binding	calf thymus DNA	54.4 µg/ml	groove binding	+	n. t.	Wang et al. 2014
chromosomal aberrations, OECD TG 473	CHL/IU cells	1.6–120 µg/ml	–m. a.: 60 µg/ml and above; +m. a.: 30 µg/ml	–	–	MHLW 2019 c, e
chromosomal aberrations, OECD TG 473	peripheral human lymphocytes	3.125–100 µg/ml	–m. a.: 150 µg/ml and above; +m. a.: 50 µg/ml and above; precipitation: 150 µg/ml and above	–	–	BioReliance 2001, pp. 340–378
recombination	Sp5 and SPD8 cell lines, derived from V79 cells (Helleday et al. 1998; Zhang and Jenssen 1992)	5–40 µg/ml	IC_50-G_: Sp5: 0.08 mM (44 µg/ml); SPD8: 0.09 mM (49 µg/ml)	–	–	Helleday et al. 1999

^a)^ The results are statistically significant unless specified otherwise.

–: negative results; +: positive results; IC_50-G_: inhibition of growth by 50%; m. a.: metabolic activation system; n. t.: not tested

Tetrabromobisphenol A yielded negative results in a test for the induction of non-reciprocal recombination (gene conversions) in the Saccharomyces cerevisiae strain D3 (SRI 1976 a, b, c). Two reviews included several studies that were carried out with Saccharomyces cerevisiae strains, but did not describe the experiments in further detail. These were probably also gene conversion tests. All tests yielded negative results (EU 2006; WHO 1995). The tests have not been included in Table 4 because they were not described in more detail.

A study that applied different spectroscopic methods revealed that tetrabromobisphenol A binds non-covalently to calf thymus DNA. A “groove binding” mechanism was determined based on molecular modelling data (Wang et al. 2014). This is not to be interpreted as direct interaction with the DNA. However, replication and transcription may be disrupted because of non-covalent binding and the filling of the grooves of the DNA’s double helix.

In 2 tests for chromosomal aberrations carried out according to OECD Test Guideline 473 in CHL/IU cells (MHLW 2019 c, e) and in peripheral human lymphocytes (BioReliance 2001, pp. 340–378), tetrabromobisphenol A did not cause clastogenicity or polyploidy.

In a test for the induction of non-reciprocal recombination (gene conversions) in the hamster cell lines Sp5 and SPD8, tetrabromobisphenol A did not cause an increase in gene conversions up to a concentration of 40 µg/ml (Helleday et al. 1999).

#### 5.6.2 In vivo

In a study that was described in detail in Section 5.2.2, oxidative stress was induced in the kidneys and testes, but not in the liver, of male juvenile Sprague Dawley rats at 500 mg/kg body weight and day. Administration by gavage for 30 days, beginning at an age of 18 days, led to an increase in the concentration of the biomarker 8-hydroxy-2′-deoxyguanosine (Choi et al. 2011).

Five male and 5 female B6C3F1/N mice were examined for the induction of micronuclei in peripheral blood erythrocytes after receiving gavage doses of tetrabromobisphenol A of 0, 10, 50, 100, 500 or 1000 mg/kg body weight and day (vehicle: corn oil) for 3 months. The percentage of normochromatic erythrocytes with micronuclei was not increased by exposure to tetrabromobisphenol A. The ratio of polychromatic to normochromatic erythrocytes remained unchanged (NTP 2014). For this reason, it cannot be determined whether the bone marrow was reached in this study. The toxicokinetics studies found that tetrabromobisphenol A is systemically available after oral administration.

### 5.7 Carcinogenicity

#### 5.7.1 Short-term studies

Offspring of F344 rats were used to study the effects of early exposure to tetrabromobisphenol A on the carcinogenicity induced by 7,12-dimethylbenz[*a*]anthracene and *N*-bis(2-hydroxypropyl)nitrosamine later in life. To expose the offspring to tetrabromobisphenol A during lactation, groups of 6 dams were given tetrabromobisphenol A with the feed up to 3 weeks after the birth in concentrations of 0 (control), 0.01%, 0.1% or 1% (about 12, 120, 1200 mg/kg body weight and day; conversion factor 0.12 (subacute) according to EFSA (2012)). During the first 2 weeks after weaning, the offspring were fed a diet containing tetrabromobisphenol A. They were then given feed that did not contain the substance for the following 4 weeks. From weeks 7 to 10, the offspring were given 0.08% or 0.2% *N*-bis(2-hydroxypropyl)nitrosamine with the drinking water (about 96, 240 mg/kg body weight and day; conversion factor 0.12 (subacute) according to EFSA (2012)) and a single gavage dose of 7,12-dimethylbenz[*a*]anthracene of 50 mg/kg body weight in sesame oil in week 7. The animals were then sacrificed and examined, the males after 37 weeks, the females after 47 weeks. The incidence of follicular thyroid adenomas was increased with statistical significance in the females treated with 1% tetrabromobisphenol A in comparison with the incidence determined in the control animals (controls: 13/22 (59%), 0.01%: 9/13 (69%), 0.1%: 11/17 (65%), 1%: 12/13 (92%)). At concentrations of 0.01% and above, the incidence of transitional cell papillomas of the bladder was increased with statistical significance in the females in comparison with the incidence found in the control group (controls: 0/23 (0%), 0.01%: 3/13 (23%), 0.1%: 4/17 (24%), 1%: 4/13 (31%)). The incidence of malignant tumours was not increased. Survival, body weights, body weight gains, and feed and water consumption were not affected by treatment (Imai et al. 2009). The findings of this study cannot be used as evidence that tetrabromobisphenol A induces carcinogenic effects that are relevant to humans because initiators were administered in high doses.

#### 5.7.2 Long-term studies

Carcinogenicity studies with tetrabromobisphenol A are shown in Table 5 and 6.

In a 2-year carcinogenicity study carried out by the NTP, Wistar Han rats were given tetrabromobisphenol A in gavage doses of 0, 250, 500 or 1000 mg/kg body weight and day. The uterus was identified as the target organ of carcinogenicity in rats. In the female animals, first a transverse section about 0.5 cm from the cervix was taken through each uterine horn. This transverse section was used for a first histopathological examination. Adenomas and adenocarcinomas were observed. A longitudinal section was then taken for a second histopathological examination. This section was taken: 1) to determine the site of origin for the tumours, 2) to fulfil the need for a complete review of the cervices for stromal hyperplasia and stromal fibrosis and 3) to look for additional neoplasms. Longitudinal sections were taken of all remaining tissues of the cervix, vagina and uterus. For uterine adenomas, a positive trend was determined in the transverse review by poly-3 test. For uterine adenocarcinomas, there was a positive trend in the transverse review and positive trends and significantly increased incidences at 500 and 1000 mg/kg body weight and day in the longitudinal and combined reviews. Uterine adenomas and adenocarcinomas were combined with malignant mixed Müllerian tumours of the uterus for statistical analysis because of the histogenesis theory and the occurrence of epithelial metastases. These show that the epithelial component is the driving force behind the formation of malignant mixed Müllerian tumours (see Section 2). The combined evaluation of adenomas, adenocarcinomas and malignant mixed Müllerian tumours in both sections as well as the combination of the sections revealed positive trends and significantly increased incidences at 500 and 1000 mg/kg body weight and day. Uterine tumour metastases were found in the following organs: intestines, liver, mesentery, pancreas, forestomach, adrenal cortex, lymph nodes, spleen, thymus, skeletal muscle, lungs, kidneys and bladder (NTP 2014). The metastatic rate for malignant mixed Müllerian tumours was 76% (4/6), while that for adenocarcinomas was 24% (11/45) (Dunnick et al. 2015). The total incidence of point mutations in exons 5-8 of the Tp53 gene was increased in uterine adenocarcinomas from rats exposed to tetrabromobisphenol A in comparison with the incidences found in spontaneous adenocarcinomas from untreated animals (10/16, 63%; controls: 1/9, 11%; silent mutations that resulted in the replacement of the synonymous amino acid were not taken into consideration but were also higher in tetrabromobisphenol A-treated rats). The adenocarcinomas from 2 rats that were exposed to tetrabromobisphenol A harboured multiple mutations (NTP 2014). In the longitudinal sections, the incidence of atypical endometrial hyperplasia in the uterus was increased with statistical significance at the low dose and above; these were regarded as preneoplastic lesions (Dunnick et al. 2015). In male rats, the incidence of adenomas in the interstitial cells of the testes was increased at the highest dose of 1000 mg/kg body weight and day. In conclusion, the occurrence of adenomas in the testes of male Wistar Han rats is regarded as equivocal evidence of the induction of carcinogenic activity by tetrabromobisphenol A. The increased incidence of epithelial tumours in the uterus (predominantly adenocarcinomas) provides clear evidence of this activity in females (NTP 2014). Atypical hyperplasias are focal, generally non-invasive changes originating in the glandular epithelium (Dixon et al. 2014). The probability of finding atypical hyperplasia is increased by including longitudinal sections because the surface area examined is larger than that of a transverse section.

Information was published by the breeding laboratory about neoplastic and non-neoplastic changes in this strain. The document does not include any data for the incidences of atypical hyperplasia, but does provide data for the incidences of endometrial adenocarcinomas. An average incidence of 2.47% (range: 0.89% to 14%) was determined for the time period between 1997 and 2009 based on the findings of 16 studies carried out at 4 different test laboratories in the US, Canada and Europe (Charles River Laboratories 2011). It is assumed that transverse sections were taken for examination because this reflects the standard procedure used during this time. Additional longitudinal sections are taken only if needed. These data were compared with the findings for endometrial adenocarcinomas obtained from transverse sections taken in the carcinogenicity study of tetrabromobisphenol A. The data for the controls and for the low dose group were in the range of the historical controls. The data for the groups given doses of 500 mg/kg body weight and day and above were outside the range of the historical controls.

The mechanisms of tumour development are described in Section 2.

In a carcinogenicity study, tetrabromobisphenol A was given to B6C3F1/N mice in gavage doses of 0, 250, 500 or 1000 mg/kg body weight and day. In the high dose group, an insufficient number of animals survived to allow for an evaluation of the carcinogenic effects. The incidence of multiple hepatocellular adenomas was increased with statistical significance in male mice at 500 mg/kg body weight and day. The incidences of hepatoblastomas and of hepatocellular carcinomas or hepatoblastomas (combined) were likewise increased in the males at 250 mg/kg body weight and day. At 250 and 500 mg/kg body weight and day, the incidences of hepatoblastomas in male mice were higher than those determined in the historical controls for studies using corn oil and all routes of administration. The combined incidences of adenomas and carcinomas of the caecum and the colon were increased in male mice at 500 mg/kg body weight and day and exhibited a significant trend. In addition, the incidences surpassed those of the historical controls for studies using corn oil and all routes of administration. A rectal adenoma was found in 1 female of the group given 500 mg/kg body weight and day. In male mice, the incidence of haemangiosarcomas was increased in all organs at 500 mg/kg body weight and day and a significant trend was evident; the same was found for the combined incidence of haemangiomas and haemangiosarcomas. However, the incidences for these tumour groups were within the range of the historical control values of the laboratory for corn oil and all routes of administration. The tumour incidence in female mice was not increased by exposure to the substance. In conclusion, the increased incidence of hepatoblastomas is regarded as some evidence that tetrabromobisphenol A causes carcinogenic effects in male B6C3F1/N mice, but there is no evidence of this effect in female animals (NTP 2014).

The International Agency for Research on Cancer (IARC) has classified tetrabromobisphenol A in Group 2A (“probably carcinogenic to humans”) (IARC 2018).

Assuming a linear dose–response relationship, a BMDL_10_ of 103 mg/kg body weight and day was determined by benchmark calculation as the starting point for the sum of adenomas, adenocarcinomas and mixed Müllerian tumours. After adjustment, a human equivalent dose of 25.3 mg/kg body weight and day was calculated following the procedure established by the US EPA. After applying the safety factor (100) that is generally used for the evaluation of environmental chemicals, a cancer safe dose (RfDCancer) of 0.26 mg/kg body weight and day was obtained, which is equivalent to a no significant risk level of 20 mg/day (assuming a body weight of 70 kg). It was not possible to calculate a BMD for hyperplasias because a dose–response relationship was not observed (Pecquet et al. 2018). The authors assumed a linear dose–response relationship, which is not correct in view of the non-genotoxic mechanism of action. However, the authors deliberately made this assumption because this represents the worst-case scenario.

**Tab.5:** Carcinogenicity study in rats with tetrabromobisphenol A

Author:	Dunnick et al. 2015; NTP 2014				
Purity:	> 99%				
Species:	**rat**, Wistar Han [Crl:WI(Han)], control group and high dose group: 60 ♂, 60 ♀, other groups: 50 ♂, 50 ♀				
Administration route:	gavage, vehicle: corn oil				
Dose:	0, 250, 500, 1000 mg/kg body weight and day				
Duration:	♂: 104 weeks, ♀: 105 weeks, 5 days/week, interim necropsy at the age of 3 months: control group and high dose group: 10 ♂, 10 ♀				
Toxicity:	**250 mg/kg body weight and above**: ♀: uterus: atypical endometrial hyperplasia (see below); **500 mg/kg body weight and above**: ♂: body weights ↓ (after 25 weeks, by at least 10%); ♀: ovaries: rete ovarii cysts; (see Section 5.2.1)				
**Parameter**	**Sex**	**Dose [mg/kg body weight and day]**			
		**0**	**250**	**500**	**1000**
surviving animals	♂	33/50 (66%)	28/50 (56%)	38/50 (76%)	39/50 (78%)
	♀	35/50 (70%)	34/50 (68%)	29/50 (58%)	33/50 (66%)
**tumours and preneoplastic lesions**:					
**uterus**:					
**transverse section**:					
endometrium, hyperplasia, cystic	♀	8/50 (16%)	13/50 (26%)	11/50 (22%)	18/50 (36%)*
adenomas	♀	0/50 (0%) p = 0.010^#^	0/50 (0%)	3/50 (6%)	4/50 (8%)
adenocarcinomas, including multiple	♀	3/50 (6%) p = 0.016^#^	3/50 (6%)	8/50 (16%)	9/50 (18%)
malignant mixed Müllerian tumours	♀	0/50 (0%)	4/50 (8%)	0/50 (0%)	2/50 (4%)
adenomas, carcinomas or malignant mixed Müllerian tumours	♀	3/50 (6%) p = 0.003^#^	7/50 (14%)	11/50 (22%)*	13/50 (26%)**
**longitudinal section**:					
endometrium, hyperplasia, cystic	♀	23/50 (46%)	30/50 (60%)	28/50 (56%)	31/50 (62%)
endometrium, hyperplasia, atypical	♀	2/50 (4%)	13/50 (26%)**	11/50 (22%)**	13/50 (26%)**
adenomas	♀	3/50 (6%)	2/50 (4%)	1/50 (2%)	3/50 (6%)
adenocarcinomas, including multiple	♀	4/50 (8%) p = 0.003^#^	9/50 (18%)	15/50 (30%)**	15/50 (30%)**
malignant mixed Müllerian tumours	♀	0/50 (0%)	0/50 (0%)	0/50 (0%)	1/50 (2%)
adenomas, carcinomas or malignant mixed Müllerian tumours	♀	6/50 (12%) p = 0.008^#^	10/50 (20%)	16/50 (32%)**	16/50 (32%)*
**transverse and longitudinal section**:					
endometrium, hyperplasia, cystic	♀	24/50 (48%)	31/50 (62%)	30/50 (60%)	32/50 (64%)
endometrium, hyperplasia, atypical	♀	2/50 (4%)	13/50 (26%)**	11/50 (22%)**	13/50 (26%)**
adenomas	♀	3/50 (6%)	2/50 (4%)	4/50 (8%)	6/50 (12%)
adenocarcinomas, including multiple	♀	4/50 (8%) p = 0.002^#^	10/50 (20%)	15/50 (30%)**	16/50 (32%)**
initial occurrence (days)	♀	713	548	321	442
malignant mixed Müllerian tumours	♀	0/50 (0%)	4/50 (8%)	0/50 (0%)	2/50 (4%)
adenomas, carcinomas or malignant mixed Müllerian tumours	♀	6/50 (12%) p < 0.002^#^	11/50 (22%)	16/50 (32%)**	19/50 (38%)**
initial occurrence (days)	♀	668	548	321	442
**testes**:					
adenomas of the interstitial cells, bilateral	♂	0/50 (0%)	0/50 (0%)	1/50 (2%)	0/50 (0%)
adenomas of the interstitial cells, including bilateral	♂	0/50 (0%) p = 0.023^#^	0/50 (0%)	1/50 (2%)	3/50 (6%)

* p ≤ 0.05;

** p ≤ 0.01; poly-3 test, after adjusting for intercurrent mortality

^#^poly-3 trend test;

Historical controls: 2-year studies, all routes of administration: malignant mixed Müllerian tumours: 0/150; adenocarcinomas uterus: 7/150 (including an endometrial carcinoma) (NTP 2014)

**Tab.6:** Carcinogenicity study in mice with tetrabromobisphenol A

Author:	Dunnick et al. 2015; NTP 2014				
Purity:	> 99%				
Species:	**mouse**, B6C3F1/N, 50 ♂, 50 ♀				
Administration route:	gavage, vehicle: corn oil				
Dose:	0, 250, 500, 1000 mg/kg body weight and day				
Duration:	105 weeks, 5 days/week				
Toxicity:	**250 mg/kg body weight and above**: ♂: liver: eosinophilic foci (see below); kidneys: cytoplasmic changes in the tubules; ♀: forestomach: ulcer, mononuclear cell infiltration, inflammation, epithelial hyperplasia; **500 mg/kg body weight**: ♂: liver: clear cell foci (see below); forestomach: ulcer, mononuclear cell infiltration, inflammation, epithelial hyperplasia; **1000 mg/kg body weight**: mortality ↑; ♀: body weights ↓ (after 25 weeks, at least 10%); animals not assessed because of high mortality (see Section 5.2.1)				
**Parameter**	**Sex**	**Dose [mg/kg body weight and day]**			
		**0**	**250**	**500**	**1000**
surviving animals	♂	33/50 (66%)	26/50 (52%)	39/50 (78%)	12/50 (24%)
	♀	40/50 (80%)	31/50 (62%)	36/50 (72%)	4/50 (8%)
**tumours and preneoplastic lesions**:					
**liver**:					
clear cell foci	♂	11/50 (22%)	10/50 (20%)	25/50 (50%)**	
	♀	3/50 (6%)	4/50 (8%)	3/50 (6%)	
eosinophilic foci	♂	20/50 (40%)	33/50 (66%)**	40/50 (80%)**	
	♀	11/50 (22%)	16/50 (32%)	11/50 (22%)	
hepatocellular adenomas, multiple	♂	12/50 (24%)	20/50 (40%)	28/50 (56%)*	
hepatocellular adenomas, including multiple	♂	32/50 (64%)	33/50 (66%)	38/50 (76%)	
hepatoblastomas	♂	2/50 (4%) p = 0.065^#^	11/50 (22%)**	8/50 (16%)	
hepatocellular carcinomas	♂	11/50 (22%)	15/50 (30%)	17/50 (34%)	
hepatocellular carcinomas or hepatoblastomas	♂	12/50 (24%) p = 0.099^#^	24/50 (48%)**	20/50 (40%)	
**caecum or colon**:					
adenomas or carcinomas	♂	0/50 (0%) p = 0.039^#^	0/50 (0%)	3/50 (6%)	
**all organs**:					
haemangiosarcomas	♂	1/50 (2%) p = 0.014^#^	5/50 (10%)	8/50 (16%)*	

* p ≤ 0.05;

** p ≤ 0.01; poly-3 test, after adjusting for intercurrent mortality

^#^poly-3 trend test

♀: no increased incidence of tumours induced by substance

historical controls, 2-year studies, 2004–2013 (NTP 2014):

hepatocellular carcinomas, including multiple:

corn oil gavage studies: 87/250 (34.8% ± 10.9%), range 22%–44%;

all routes of administration: 348/949 (36.7% ± 11.4%), range 22%–56%;

hepatoblastomas:

corn oil gavage studies: 9/250 (3.6% ± 2.6%), range 0–6%;

all routes of administration: 40/949 (4.2% ± 3.5%), range 0–12%;

hepatocellular carcinomas or hepatoblastomas:

corn oil gavage studies: 93/250 (37.2% ± 10.0%), range 24%–48%;

all routes of administration: 371/949 (39.1% ± 11.6%), range 22%–54%

haemangiosarcomas:

corn oil gavage studies: 28/250 (11.2% ± 6.4%), range 2%–18%;

all routes of administration: 92/980 (9.7% ± 4.5%), range 2%–18%

## 6 Manifesto (MAK value/classification)

After repeated administration by gavage, the target organs were the endocrine system in male and female rats with a decrease in the total T4 concentration, and the kidneys in male mice with cytoplasmic changes in the tubules. Tetrabromobisphenol A induced uterine tumours in female Wistar Han rats as well as hepatoblastomas and haemangiosarcomas in male B6C3F1/N mice.

**MAK value and peak limitation. **Studies with test persons or epidemiological studies are not available.

A NOAEL of 50 mg/kg body weight and day was derived for the most sensitive end point, a decrease in the T4 concentration at doses of 100 mg/kg body weight and day and above, from a 14-week study with gavage administration in male and female F344/NTac rats (NTP 2014). The cytoplasmic changes found in the renal tubules at doses of 500 mg/kg body weight and day and above were identified as the critical end point of the 14-week gavage study with male B6C3F1/N mice. The NOAEL was 100 mg/kg body weight and day (NTP 2014).

A NOAEL for effects on the uterus was not derived for Wistar Han rats from the findings of the 2-year study, only a LOAEL of 250 mg/kg body weight and day for atypical hyperplasia (Dunnick et al. 2015; NTP 2014). Histological examination of the uterus did not reveal any effects up to a dose of 1000 mg/kg body weight and day in rat studies with shorter periods of exposure of up to 14 weeks or in the carcinogenicity study in Wistar Han rats at interim necropsy after 3 months (Cope et al. 2015; NTP 2014; Osimitz et al. 2016). Furthermore, no NOAEL for effects on the liver was derived from the long-term study in mice, only a LOAEL of 250 mg/kg body weight and day for eosinophilic foci (Dunnick et al. 2015; NTP 2014). A NOAEL of 100 mg/kg body weight and day was the lowest NOAEL derived from short-term studies for effects on the liver. This NOAEL was determined by a 14-week study for an increase in liver weights in male mice (an increase of less than 20% is not adverse) (LOAEL 500 mg/kg body weight and day; NTP 2014). Histological examination of the liver did not reveal any unusual findings up to a dose of 1000 mg/kg body weight and day, the highest dose tested, in short-term studies and in the long-term study at interim necropsy (NTP 2014).

The following toxicokinetic data are taken into consideration for the extrapolation of the NOAELs of 50 mg/kg body weight and day for rats (T4 concentration) and 100 mg/kg body weight and day for mice (kidneys) to a concentration in workplace air: the corresponding species-specific correction values for the rat and mouse (1:4 and 1:7, respectively), the experimentally determined oral absorption (72%; Hakk et al. 2000), the body weight (70 kg), the respiratory volume (10 m^3^) of the person and the assumed 100% absorption by inhalation. Other factors that are taken into consideration are the extrapolation of the data from animal studies to humans (1:2), no time-extrapolation for the reduced T4 concentrations and the study duration of 14 weeks (1:2) for the effects on the kidneys because the effects in the 2-year study occurred at the lowest dose of 250 mg/kg body weight and day and above (NTP 2014). On this basis, a concentration in air of 32 mg/m^3^ has been calculated using the data from rats and of 18 mg/m^3^ using the data from mice, which would result in a limit value in air for the inhalable fraction of tetrabromobisphenol A of 10 mg/m^3^.

However, the adenocarcinomas of the uterus and their precursors must likewise be taken into consideration. The very low effect concentrations in vitro at possible cellular points of action, the inhibition of the SULT1E1 sulfotransferase that metabolizes oestradiol (IC_50_ 12 to 33 nM; Hamers et al. 2006; Kester et al. 2002) and the possible proliferative effects via GPER at 10 nM (Hoffmann et al. 2017 b) suggest that tetrabromobisphenol A may already induce effects in vivo at concentrations below 10 mg/m^3^. There are no in vivo correlates for these mechanisms. A NOAEL is not available for the development of preneoplastic lesions (atypical hyperplasias) that were observed in Wistar Han rats at doses of 250 mg/kg body weight and day and above (Dunnick et al. 2015; NTP 2014). The course of the dose–response curve is not known. The extrapolation of the LOAEL of 250 mg/kg body weight and day for atypical endometrial hyperplasias to a concentration in air results in a value of 157 mg/m^3^ based on the same assumptions as used above. In view of the severity of the effects (preneoplastic lesions), the margin between the LOAEL and the concentration in air of 10 mg/m^3^ is not considered adequate. As a result, and because of the uncertainties relating to the dose–response curve, a MAK value has not been derived based on the LOAEL for atypical hyperplasia.

A MAK value cannot be derived because the data needed to establish a NOAEL for atypical hyperplasia are not available and it is neither possible to clarify whether the uterine tumours are strain-specific effects nor to characterize the mechanism of action of tumour formation in greater detail.

As a MAK value has not been derived, peak limitation does not apply.

**Prenatal toxicity. **In a prenatal developmental toxicity study carried out according to OECD Test Guideline 414 with gavage administration in rats from gestation days 0 to 19, developmental and maternal toxicity were not observed up to the highest dose tested of 1000 mg/kg body weight and day. The NOAEL for developmental toxicity was 1000 mg/kg body weight and day, the highest dose (Cope et al. 2015; MPI Research 2001). A NOAEL of 1000 mg/kg body weight and day, the highest dose, was derived for perinatal toxicity from the findings of a 2-generation study with gavage administration in rats (Cope et al. 2015).

As no MAK value has been derived, the substance has not been classified in a pregnancy risk group.

**Carcinogenicity. **The uterus was the target organ of carcinogenicity in Wistar Han rats. The incidence of adenocarcinomas was increased in the 2-year carcinogenicity study at 500 mg/kg body weight and day and above. In addition, malignant mixed Müllerian tumours, a very rare form of tumour, occurred in isolated cases and uterine tumour metastases were found in several organs. Atypical endometrial hyperplasia, a preneoplastic lesion, was observed at the lowest dose of 250 mg/kg body weight and day and above. No carcinogenic effects were found in male rats (Dunnick et al. 2015; NTP 2014). In the 2-year carcinogenicity study, the incidence of hepatoblastomas and the combined incidence of hepatocellular carcinomas and hepatoblastomas were increased with statistical significance in male B6C3F1/N mice at 250 mg/kg body weight and day, but not at 500 mg/kg body weight and day. At both doses, the incidences for hepatoblastomas were higher than those of the historical controls. In male mice, the incidence of haemangiosarcomas (all organs) and the combined incidence of haemangiomas and haemangiosarcomas were increased at 500 mg/kg body weight and day with a significant trend. However, the incidences were within the range of those of the historical controls of the laboratory. The incidence of tumours in female mice was not increased (NTP 2014). Tetrabromobisphenol A was carcinogenic in female Wistar Han rats and in male B6C3F1/N mice.

There is no evidence to suggest that the substance is genotoxic (see below). Non-genotoxic mechanisms are responsible for tumour formation. The development of uterine tumours in Wistar Han rats is attributed to oestrogenic and non-oestrogenic effects. A gene expression study in female Wistar Han rats demonstrated that tetrabromobisphenol A disrupts oestrogen homeostasis; this is not mediated by an agonist effect on the oestrogen receptors ERα and ERβ (Sanders et al. 2016). The proliferative effects on the endometrium are attributed to oestrogen (NTP 2014). The non-oestrogenic effects include immunosuppressive effects. As there is no evidence that the uterine tumours are specific to a certain strain, the tumours have been included in the evaluation in compliance with the procedure established by the Commission (Laube et al. 2019). In the B6C3F1/N mouse strain, hepatoblastomas are known to develop in male mice via a CAR/PXR induction mechanism. The eosinophilic foci are indirect precursors that lead to hepatoblastomas via hepatocellular carcinomas. The Commission assumes that the tumour is relevant to humans, but that humans are much less susceptible (Felter et al. 2018). It is postulated that, besides PPARγ agonistic effects, the development of haemangiosarcomas in male B6C3F1/N mice is associated with the comparably higher basal proliferation rate of endothelial cells in this strain of mouse in comparison with the rates found in F344 rats and humans. There is no evidence that the effects are species-specific; the Commission therefore assumes that the tumour is relevant to humans.

Due to the non-genotoxic mechanism of action, it is assumed that a NAEL (no adverse effect level) can be established for the carcinogenic effects. For this reason, it would be possible to classify tetrabromobisphenol A in Carcinogen Category 4. As the available data are not sufficient to allow for the derivation of a MAK value (see above), the substance has been classified in Carcinogen Category 2 according to the procedure currently followed by the Commission (Hartwig and MAK Commission 2022) and bears the footnote “Prerequisite for Category 4 in principle fulfilled, but insufficient data available for the establishment of a MAK or BAT value”.

**Germ cell mutagenicity. **No studies with germ cells are available. Tetrabromobisphenol A was not mutagenic in numerous bacterial gene mutation tests (EU 2006; Litton Bionetics Inc. 1976, pp. 214–223, 1977, pp. 21–32; MHLW 2019 b, e; Mortelmans et al. 1986; NTP 2014; SRI 1976 a, b). Tetrabromobisphenol A did not cause clastogenicity or polyploidy in 2 tests for chromosomal aberrations in mammalian cells (BioReliance 2001, pp. 340–378; MHLW 2019 c). Oxidative stress in the kidneys and testes of male juvenile Sprague Dawley rats was observed at a tetrabromobisphenol A dose of 500 mg/kg body weight and day (Choi et al. 2011). Tetrabromobisphenol A was not clastogenic in male and female B6C3F1/N mice after exposure for 3 months to doses up to 1000 mg/kg body weight and day (NTP 2014). According to the available data, tetrabromobisphenol A is not genotoxic. For this reason, the substance has not been classified in a category for germ cell mutagens.

**Absorption through the skin. **Tetrabromobisphenol A causes low acute toxicity after dermal application. Data from in vitro and in vivo studies are available for the absorption of tetrabromobisphenol A through the skin. In vitro studies carried out by Knudsen et al. (2015) demonstrated that the barrier properties of human skin are markedly better than those of rat skin.

Three in vivo studies investigated the penetration of tetrabromobisphenol A through the rat skin. On the basis of the penetration data reported by the studies of Yu et al. (2016, 2017) and Knudsen et al. (2015), 0.3 mg to 361.9 mg of tetrabromobisphenol A would be absorbed after 1-hour exposure of 2000 cm^2^ of skin. The amount is dependent on the dose and sex.

Data from in vivo studies suggest that relevant amounts of the substance are absorbed through the skin. As a result, the substance has been provisionally designated with an “H” (for substances which can be absorbed through the skin in toxicologically relevant amounts) because it is classified as a Category 2 carcinogen, but there is currently no known threshold for the carcinogenic effects.

**Sensitization. **There are no findings of sensitizing effects in humans and no positive results from experimental studies with animals or in vitro studies. Therefore, tetrabromobisphenol A has not been designated with “Sh” or “Sa” (for substances which cause sensitization of the skin or airways).
